# The Law Behind Dispute Onset: How Legal Uncertainty Drives Maritime Boundary Disputes

**DOI:** 10.1177/00220027241305076

**Published:** 2024-12-05

**Authors:** Umut Yüksel

**Affiliations:** 1 Department of Political and Social Sciences, 16770 Universitat Pompeu Fabra, Barcelona, Spain; 2 Global Governance Centre, 30525 Geneva Graduate Institute, Geneva, Switzerland

**Keywords:** international law, international security, maritime boundaries, dispute onset

## Abstract

The making of international law through multilateral conventions and adjudication often leads to periods of legal uncertainty, times in which there are alternative rules and divergent views on how they ought to be applied to particular cases. I argue that legal uncertainty gives states opportunities and incentives to formulate excessive unilateral claims, thus making disputes more likely to arise. I illustrate my argument with a comprehensive analysis of maritime boundary disputes in the aftermath of the Second World War. In this period, the law regulating maritime boundary-making has been marked by varying degrees of uncertainty due to different rules and interpretations proffered by various multilateral and judicial lawmaking attempts. I find strong evidence that high legal uncertainty is associated with an increased probability of dispute onset. The analysis calls for an important rethinking of the impact of legalization on international affairs, both in maritime boundary-making and in other issues areas.

## Introduction

The use of international law to regulate international affairs has become common in several domains of global politics (Goldstein et al. 2000). Scholars have argued that legalization, especially in its “hard” forms with written rules and delegated authority for the resolution of disputes over their interpretation, can help states manage their disagreements through specific legal proceedings or in the shadow of plausible judicial decisions (Abbott and Snidal 2000, 431). While compliance scholars have shown that law’s effect may be uneven (see, e.g., Hafner-Burton and Tsutsui 2007), the common expectation that legalization should contribute to dispute avoidance and negotiated settlements remains pervasive. The expectation that international law serves to reduce disputes is echoed in international relations and legal scholarship alike (for a critique of this shared assumption, see Hakimi 2017).

My account shows that international law can often foster new disputes. Specifically, international lawmaking through multilateral treaties and by way of adjudication can sow the seeds of new disputes by providing alternative rules and interpretations to states and increasing the complexity of applying law to specific cases. Without a central authority that can decisively establish what the law is at any moment, new rules and interpretations do not immediately supersede the old ones, adding instead to the range of rules, understandings, and acceptable practices that states can choose from. Especially if different rules and interpretations advantage states differently, states have incentives to adopt policies on the basis of rules that are the most favorable to them. This can lead to positions that may clash with those of other states with similar motivations. Thus, legal uncertainty created in the course of lawmaking can make disputes more likely to arise in the first place.

I discuss how international lawmaking generates legal uncertainty and test how the latter influences dispute onset in the domain of maritime boundary-making in the aftermath of the Second World War. This area provides a good testing ground because there has been a great increase in the extent of unilateral state claims^1^ in the sea and the law of the sea evolved considerably through multilateral negotiations and adjudication parallel to this expansion of state jurisdiction. While states traditionally claimed sovereignty over limited maritime areas, often reaching no more than 3 nautical miles (nm) from their coasts, they began to assert exclusive rights over ever greater areas and for an increasing range of uses (Schofield 2012). Several international conferences were held to codify this new law of the sea, often outpaced by the evolving state practice (Rosenne and Gebhard 2008).

Between unilateral claim-making and multilateral lawmaking, bilateral state activity flourished over areas where more than one state could assert jurisdiction. Several pairs of states finding themselves contiguous around such areas managed to agree on the course of their common maritime boundary without much difficulty. For instance, France and Australia delimited their maritime boundaries around New Caledonia after only 3 days of negotiations in 1982 (Prescott 1993, 1187). Conventional wisdom and existing international relations scholarship hold that legalization was a success story in the field of the law of the sea, with the 1982 UN Convention on the Law of the Sea (UNCLOS) receiving special credit for clarifying the law. Yet, as rules were being codified, several states also entered into disputes, many of which, such as those in parts of the Mediterranean and the South China Sea, remain unresolved, four decades after the entry into force of the UNCLOS.

This paper offers a new theory about how international lawmaking can as easily drive new disputes as it can help prevent them. Treating boundaries as institutions that generate joint gains for those that manage to agree on them (Simmons 2005), it rests on a set of assumptions that hold that each state is interested in maximizing the area under their control, but each would rather avoid costly conflict and negotiate to divide areas that potentially overlap. Importantly, I show how the legal nature of maritime boundary claims present two additional constraints that have not been sufficiently appreciated. The first is the *desire for recognition*, which is in harmony with the desire to avoid conflict and pursue joint gains. The second is the principle of *equality of states* before the law, which means that a state cannot claim a right by virtue of an international legal entitlement if it is not willing to concede the same right to others.

The legal origin and nature of maritime boundaries and the desire for legal recognition of claimed maritime jurisdictions lead states to resort almost exclusively to international law as they justify their claims. To understand the patterns of claim-making and maritime boundary disputes, it is thus crucial to consider what international legal rules have to say about the acceptable extent of state jurisdiction in the sea and the appropriate ways to delimit overlapping boundaries. I show that this body of law has been subject to intense debates during the second half of the twentieth century, when authoritative legal sources were either silent on key aspects of maritime boundary-making or disagreed over what the law is. I argue that these disagreements both reflected and further contributed to *international legal uncertainty*, which played an important role in leading states into new disputes.

I define international legal uncertainty as the degree of disagreement among authoritative sources of international law as to what the law requires. Legal uncertainty is high when sources disagree, and low when they agree. While it is useful to conceptualize and analyze legal uncertainty in a field where international law matters and a legal justification is required for claims, legal uncertainty can well be present in different regimes in varying degrees. The theory I provide can be relevant to understanding how states take positions and make claims on a variety of issues, ranging from international economic policy and human rights to laws of war.

All other things equal, a higher degree of legal uncertainty should give states opportunities and incentives to make maximalist claims that can drive dispute onset. This is mainly because different states are likely to be advantaged by different competing rules and interpretations, and each will pick the rule or interpretation that maximizes its maritime area at the expense of its neighbors. I test this claim on an original dataset that contains all the pairs of coastal states that have had maritime boundaries to delimit in the post-World War II period. The statistical analyses provide strong evidence that legal uncertainty is associated with increased probability of dispute onset, consistent with my expectations. I subject this result to a series of robustness checks and adduce additional evidence regarding the suggested mechanism by which uncertainty should affect dispute onset.

This paper advances scholarship by showing that lawmaking processes and outcomes may have effects that are the opposite of what many understand to be the conflict prevention function of law. Making of new laws and interpretation of legal rules by institutions can foster new conflicts, as new rules and interpretations create alternative sticking points to justify conflicting claims. This understudied but plausible effect of legalization promises to open avenues for scholars of international relations and law to examine the conditions under which lawmaking leads to conflict rather than cooperation.

## Drivers of Maritime Boundary Disputes

In the past decades, scholars have provided insights on maritime disputes as distinct from territorial ones, mainly led by the Issue Correlates of War (ICOW) project (Hensel and Mitchell 2017).^2^ Relevant recent work shows that states can enter into disputes to further their territorial ambitions or due to security or legal uncertainties stemming from the absence of clear rules about how states ought to draw their boundaries in the early decades of the post-World War II era. Another finding links uneven naval capabilities and the retreat of U.S. naval power to new maritime disputes (Mitchell 2020). Scholarship on the origins of territorial disputes identify proximity of the current boundary to historical boundary locations as conducive to novel disputes (Abramson and Carter 2016) and times of systemic uncertainty—when a regional great power is in crisis—as periods of high risk of dispute onset (Abramson and Carter 2021). I contribute to this work by theorizing legal uncertainty as a source of systemic instability that affects the incentives and opportunities of states in ways that make disputes more likely.

Several scholars have proposed that international legal rules and judicial bodies help states resolve their disputes (Ginsburg and McAdams 2004). In the context of territorial disputes, international law has been shown to aid states come to similar views as to which legal principles are relevant to their dispute and to provide states with focal points that help leaders’ expectations converge. Huth and co-authors have usefully articulated the conditions under which international law can play such a role—one of which is that the law is clear enough so that it gives rise to coherent expectations (Huth et al. 2011, 2013). Studies touching upon maritime boundary-making also concede that neither conflict nor cooperation over maritime boundaries takes place in a vacuum, due to the salience of the international law of the sea. By and large, such studies have focused on one specific institution—the 1982 UNCLOS, finding, for example, that joint membership in UNCLOS is associated with peaceful settlement attempts (Nemeth et al. 2014). According to by Ásgeirsdóttir and Steinwand (2018), UNCLOS provides the median line as a way of delimiting maritime boundaries as an “inside option” to which states could default unless bargaining asymmetries justified deviating from it. Mitchell and Owsiak (2021) find that the signature of UNCLOS is associated with a lower likelihood of new maritime disputes. Moreover, they suggest that the jurisprudence developed by various courts and tribunals allows states to anticipate what a judicial settlement would look like, which should allow them to bargain in the shadow of the law. Other scholars have contested the view that UNCLOS brought any certainty to maritime delimitation, pointing out the vagueness of its delimitation provisions, while conceding that the case law may have made the delimitation exercise more predictable in more recent years (Rothwell 2021).

Generally, the scholarship on international law and boundary making has focused on the pacifying and coordinating effect of international law. It has paid less attention to what could happen when law is unclear or able to support various conflicting claims. Mitchell (2020, 647) proposes that while uncertainties are reduced after UNCLOS is signed, states can still disagree on the basis of economic inequities that can arise due to a strict application of UNCLOS. The idea that lawmaking itself may be a source of disputes has already been suggested by scholars dealing with the impressive development of the law through international treaties in the domain of the law of the sea. Buzan (1978) identifies possible sources of disputes rooted in the comprehensive law of the sea treaty as it was being negotiated. Song and Tønnesson (2013) similarly consider the impact of the negotiations of the UNCLOS—which lasted from 1973 to 1982—on the disputes in the South China Sea, concluding that multilateral treaty-making made maritime boundaries salient to littoral states and encouraged new claims that often conflicted with each other. Wilson (1979) goes as far as to suggest that the Aegean dispute between Greece and Turkey was wholly generated by the multilateral negotiations surrounding the law of the sea regime.

While these studies provide convincing mechanisms through which multilateral treaty-making can generate new disputes, they have not tested their claims systematically. Moreover, the exclusive attention paid to the UNCLOS by most scholars left several legal developments occurring through the customary law and judicial rulings outside the picture. Recent work shows that judicial decisions incongruent with existing law and inconsistent with earlier decisions can have a defocalizing effect on state policies, with the result that state positions become more diverse rather than being unified (Yildiz and Yüksel 2024). Yet the link between diversity of state positions and actual disputes have not been explored. Overall, we still know little about the effect of the law on dispute onset, a gap that this paper begins to fill.

## Theory

### The Making of a Maritime Boundary Claim

Maritime boundary-making is the process by which states draw boundaries between their maritime zones on the one hand, and the high seas and/or the maritime zones of their neighboring states on the other hand. The process often involves two stages: a *unilateral claim* stage, and a *delimitation* stage. First, states make unilateral claims to maritime zones adjacent to their coasts where they can assert jurisdiction. Second, if a state’s claim reaches areas where a neighboring state can plausibly make a claim, the maritime zones between the states need to be delimited such that a maritime boundary is drawn to divide states’ respective areas of jurisdiction. To illustrate, the US and Mexico unilaterally extended their maritime jurisdictions to 200 nm in 1976, following which they exchanged notes to delimit a new boundary in the area resulting from these extensions (Sepúlveda 1983, 159-160).

Coastal states^3^ make unilateral claims over maritime areas with a view to furthering the attainment of their goals as they define them. These goals may relate to security, economic activities, and navigational freedoms. Expected gains of exclusive jurisdiction include benefits from the exploration and exploitation of natural resources in the areas under such control. If we grant that states benefit from establishing exclusive jurisdictions over more extensive areas rather than narrower ones, it seems quite likely that unilateral claims overlap, and maritime boundary disputes abound. This does not happen for two key reasons. First, benefits associated with establishing exclusive jurisdiction over an area and engaging in fruitful economic and other activities therein are fully realized only if a state’s claims do not overlap with its neighbors’ claims and are recognized by third states—if not by all other states, at least by an important subset of them.^4^ A possible extent to which a state would go in its claim is to make one that could get recognition from a significant number of states, including its neighbors. Recognition is especially relevant in the case of maritime boundary claims, which are new, legal claims, made over areas that were customarily thought to lie outside state jurisdiction. This important feature of modern maritime boundary claims, which also distinguishes them from claims over territory, is aptly captured by a legal scholar:

Although […] each state has a right to legislate with respect to its own territory, a unilateral extension by municipal law of the limits of its territorial sea into the high seas that are common to all and the consequent reduction of the high seas area, or the establishment of an exclusive fishing zone in the high seas to try to exclude vessels of foreign nationals, will not be valid in international law unless and until it is recognized by other states (Dean 1960, 279).

Second, a state’s claims are further constrained by the extent to which it is willing to recognize similar claims that other states will make. Indeed, when a state claims that its jurisdiction extends to a certain limit, it must admit that other states are also entitled to making claims extending to that distance.^5^ This is because the backdrop to modern maritime boundary claims was a shared and widely accepted custom regulating the limits of state jurisdiction in the sea.^6^ Moreover, at the time when state claims began to expand, the principle of sovereign equality between states was enshrined in the UN Charter. Both their origin as a modification of customary law and their appearance in the context of an international system where states were nominally equal made it hard for states to claim exceptional rights for themselves that they would deny other states.

Thus, we can propose that a coastal state will make a maritime boundary claim (1) that will give them jurisdiction over as great a maritime area as possible, (2) that will be recognized by other states, and (3) to the extent that it is willing to recognize similar claims made by others. Hence the contradictory forces exerted by states’ “interest in maximizing claims” and their interest in recognized boundaries in how they formulate maritime boundary claims (Oxman 1993, 15). Although states desire to establish exclusive control over as large a maritime area as possible, they are constrained by the need to agree both on a set of maximum outer limits to which all states would be entitled and on the specific course of maritime boundaries between themselves and their neighbors with overlapping entitlements.

Given these incentives and constraints, international law serves states as an important tool to formulate and justify claims to maritime zones as they seek the recognition of their claims and challenge the claims of others. The next section shows how lawmaking fostered diverging opinions about the extent and nature of state jurisdiction in the sea and how maritime delimitation between states should be carried out. The degree to which opinions diverged in the long process of lawmaking is what I identify to be an important factor in how states formulated new claims and entered into new disputes.

### Legal Uncertainty and Dispute Onset

Maritime boundary-making can be seen as a distribution problem in which neighboring states bargain over the division of a maritime area between them. States find themselves in a situation where they will need to agree on a common boundary almost mechanically where they are adjacent to each other or close enough to have overlapping entitlements. From then on, outcomes marked by conflict (i.e., a boundary dispute) or cooperation (e.g., a delimitation agreement) arise from a particular balance between states’ desire to delimit boundaries and that of maximizing their jurisdiction. Legal uncertainty plays an important role in the realization of both outcomes, especially that of driving new disputes.

I define *international legal uncertainty* as the degree of disagreement as to what the international law requires, rooted in the conflicting answers different sources of law give to questions about what is prescribed by the rules governing the conduct of actors. It manifests itself as conflicting interpretations, rules, or practices relevant to a particular set of activities. When it comes to activities involved in maritime boundary-making, legal uncertainty takes the form of disagreements on the *extent and nature of coastal state jurisdiction in the sea* and the *method of maritime boundary delimitation* when states have overlapping entitlements. The former includes disagreements about what the maximum extent of the territorial sea and various other maritime zones should be and what sorts of rights states should have in those zones. The latter comes into play when two states are proximate enough that they could enter into a boundary dispute or sign a delimitation agreement, and the question is that of identifying the rules that should govern the delimitation of their boundaries.

Uncertainty as it is defined here does not simply mean the lack of a treaty codifying the law in clear terms, which is what is implied in many of the studies that proxy certainty with the UNCLOS or its binding force either in general (when it is in force) or in a specific dyad (when both sides have ratified it). Neither does the proposed conception of uncertainty mean that a specific border is not delimited, or that states disagree about how it may have been delimited in the past (Goemans and Schultz 2017). Rather, uncertainty is a systemic feature that concerns the degree to which rules and interpretations relevant to boundary-making, enunciated by various sources of international law, are consistent with each other. In this sense, it is more akin to the concept of legal clarity proposed in Huth et al. (2011). Clarity of the legal principles in that context is one of the conditions put forth for a focal point to emerge. Such clarity hinges on consistent interpretations by various sources of international law, including international courts and tribunals, and it can vary over time. My concept does not contradict this idea, but further elaborates on its implications by showing that “legal principles that are subject to considerable debate and conflicting interpretations” are not only “unlikely to be helpful in resolving disputes peacefully,” as the authors suggest (Huth et al. 2011, 421), but also likely to engender new disputes.

My main contention is that legal uncertainty provides states opportunities and incentives to make extensive claims and take issue with others’ competing claims. When the law is uncertain, various rules and interpretations are available for states as they decide on their claim. A state can thus base its claim on a rule or an interpretation that supports a broader entitlement for itself than other rules and interpretations would. Such claims are more likely to reach areas where another state, relying on another available rule or interpretation, may want to assert a boundary claim.

An illustration is provided by France and Canada in the maritime area between St. Pierre and Miquelon and Newfoundland. A dispute arose in 1966 over how the boundary between these French islands off the coast of Canada and the Canadian mainland should be drawn. France supported the drawing of an equidistant median line between the islands and the Canadian mainland. Canada, for its part, could use a clause in the 1958 Continental Shelf Convention (CSC) to argue that equidistance is inappropriate because of special circumstances. The development of the law thereafter provided new ways for Canada to justify its objection to France’s claim: the ICJ’s 1969 *North Sea* case allowed it to argue that delimitation should follow equitable principles and the projection of Canada should not be cut off by giving full effect to the French islands. Canada could also argue, after the ICJ’s 1985 *Libya/Malta* case, that giving islands full effect would create disproportionate results, given that the Canadian coastline is much longer than the coastlines of the French islands facing it (Schneider 1998). As this illustration suggests, legal uncertainty created by multilateral and judicial lawmaking can provide opportunities for states to adopt conflicting positions and insist on them.

In addition to the opportunities in terms of existence of rules and interpretations accommodating a range of potentially conflicting claims, legal uncertainty also provides states *incentives* to make a claim based on the rule or interpretation that will help them maximize their maritime jurisdiction. Such incentives arise for two main reasons. First, anticipating negotiations and possible concessions, states may find it reasonable to begin with an initial claim that is more extensive than their perceived entitlement or what they would be willing to settle for. In times of legal uncertainty, states will find rules and interpretations that allow them to add to their initial claim further room for subsequent concessions. Moreover, in the strategic setting of maritime delimitation, each state should expect that its neighbors are making initial claims that are more extensive than what they are ready to settle for, which gives them incentives to counter with an extensive claim on their end.

Second, states may want to use rules or interpretations that give them the broadest area as a way of increasing the chances of those rules and interpretations to crystallize as the law. In times of legal uncertainty, states can hope to shape the law through their practice, expecting that, if enough other states follow, the practice may become generalized enough to reach a customary law status or tilt the balance towards their favored interpretation in an international treaty-making conference. Uncertain law is malleable not only in the sense that it permits the use of multiple rules as justifications, but also because how states or groups of states take position with regard to those rules will decide which rule may one day become dominant. By adopting the most beneficial rules and interpretations out of a set of competing rules and interpretations, states may reasonably seek to influence where the law is headed and where it will settle.

Given these opportunities and incentives, it is plausible that legal uncertainty leads to dispute onset, to the extent that uncertainty makes each state more likely to find a rule or an interpretation that will be more favorable to it at the expense of its neighbor. Based on this, the main hypothesis this study seeks to test is the following:

H1 Legal uncertainty increases the likelihood of maritime dispute onset.

This hypothesis can be broken down into two in terms of its observable implications, corresponding to two types of legal uncertainty, as will be discussed later. Insofar as legal uncertainty is a feature of a period of time characterized by disagreement in sources of international law, we can propose the following hypothesis:

H2 Periods with higher levels of legal uncertainty increase the likelihood of maritime dispute onset.

Insofar as legal uncertainty is more relevant to some pairs of states due to the presence of certain dyad-specific features, we can expect the following:

H3 Dyads that have features marked by legal uncertainty are more likely to experience maritime dispute onset.

The next section deals with the construction of the dataset I use to test these expectations, discusses the operationalization of legal uncertainty in both its baseline and dyad-specific components, and lays out the estimation strategy.

## Data and Methodology

To study dispute onset, it is necessary to first identify the set of pairs of states that have the possibility of entering into a maritime boundary dispute. I employ an original dataset that covers such pairs. An observation for a pair of states in a given year appears when the states composing the dyad can reasonably be expected to have a common maritime boundary to delimit. In the Online Supplement (OS), Section S1, I discuss how I decide on what is a *delimitation-relevant dyad* that needs to agree on common maritime boundaries and hence risks entering into maritime boundary disputes. The resulting dataset consists of dyads that were at risk at some point between 1946 and 2016, yielding 444 unique dyads and 18,999 dyad-year observations.

### Dependent Variable: Dispute Onset

Dispute onset is observed when two neighboring, delimitation-relevant states find themselves explicitly disagreeing over where their common maritime boundaries should lie. What triggers onset is usually an official state protest over the extent of a neighbor’s unilateral claim, or a new, actively pursued claim of one state that clearly overlaps with an earlier one made by a neighboring state. I code the year in which a dispute begins using existing datasets complemented by original research. The most relevant existing dataset of maritime disputes is the maritime claims dataset provided by the Issue Correlates of War (ICOW) Project.^7^ In order to have comprehensive information on the ways in which all relevant dyads have dealt with their maritime boundaries, I rely on various sources, including the *International Maritime Boundaries* (IMB) volumes,^8^ the UN Law of the Sea bulletins,^9^ legislation and treaty information from the UN Division of Ocean Affairs and the Law of the Sea,^10^the *Maritime Claims Reference Manual* provided by the US Department of Defense,^11^ *Limits in the Seas* volumes provided by the US Department of State,^12^ and news reports as well as case studies from the International Boundaries Research Unit (IBRU),^13^ and the CIA World Factbook.^14^

I look at dispute onset only and as long as there is such a risk. In addition to using a dataset that only focuses on the dyads which can potentially enter into a maritime boundary dispute, I consider that the risk ends after two states *fully delimit* the maritime boundary between them. Note that the bar for achieving full delimitation is high—it is not enough that an agreement delimiting the entire boundary between the two states is signed; each concerned state must also have ratified the agreement, and the agreement should thus be in force. At such a stage, we should expect boundary treaties to be especially sticky and hard to revise. In principle, a drawn maritime boundary cannot be unilaterally modified or renounced by either of the parties. Change of circumstances that can warrant a state to exit from a treaty does not apply to boundary treaties according to the Vienna Convention on the Law of Treaties (art. 62, para. 2).^15^ In practice, at least as far as delimited maritime boundaries are concerned, states do not go beyond the boundary lines they have already agreed to and initiate new conflicting claims. Indeed, I could record no instance of a new dispute onset after full delimitation. For these reasons, the analyses exclude dyad-year observations after full delimitation.

### Independent Variable: Legal Uncertainty

To measure legal uncertainty, I consider the degree to which agreement existed with regard to (1) the *extent of maritime zones* (in terms of their outer limits) and (2) the *maritime delimitation methodology* that should be used to delimit areas where more than one state can have an entitlement. Studying only the most relevant sources—multilateral treaties, custom, and judicial decisions—I assess the extent to which the views identified in these sources are consistent with each other on these two points. I also consider the views of legal scholars and practitioners about the degree of congruence among the responses given by different sources of international law. Finally, I rely on state practice as assessed by scholars and practitioners in times when there are no established treaties, custom, or case law. In theory, scholars and practitioners could fundamentally disagree about how to read the sources and state practice, as well as how to assess the state of the law. In practice, we find that legal scholars often come to similar conclusions about how the law of maritime delimitation developed, which important treaties or decisions made it more uncertain, and how the uncertainty evolved over time.

It is important to note that the diversity of responses given on these points by different sources of international law over time often create new alternatives to choose from instead of overruling earlier expressions. For instance, international courts and tribunals are often eager to appear not to overrule precedent even when they deviate from them (Guillaume 2011). Even if there was a clear hierarchy among the sources of international law, and a doctrine of binding precedent, nothing stops states from selectively citing favorable rules, interpretations, and precedents. State claims can become less credible if they are based on outdated treaties or precedent at a time when international legal sources are more consistent with each other. Yet, the expectation that an earlier, favorable understanding will be resuscitated by a treaty or a ruling may be enough for some states to use it as a justification for an expansive claim.

While all coastal states are affected by legal uncertainty to some extent, certain states or dyads have to endure it more acutely than others. I distinguish between a general, *baseline uncertainty* and a *dyad-specific uncertainty*. The former applies to all dyads with common boundaries to delimit. The latter applies to certain dyads with particular elements that are subject to disagreements beyond those that are implied by baseline uncertainty. These elements are specific geographic features near the delimitation area as well as the overall coastal configuration around it. I discuss how I operationalize these components of uncertainty in turn.

#### Baseline Legal Uncertainty

The degree to which there are different responses to the fundamental questions of maritime delimitation emanating from treaty law and judicial decisions determine the degree of disagreement within and across the sources of law. Each new response provided to these questions by an authoritative source of law has the potential to unsettle the existing level of agreement or coherence. This is why I focus on instances where the production of new responses can be expected as potential cut-off points for baseline uncertainty, around which the degree of disagreement varies.

One category of potential cut-off points includes *moments states come together to codify and further develop the international law of the sea* (such as the three UN Conferences on the Law of the Sea). Around such moments, we can reasonably expect disagreements to arise about what the current law is, how it is changing, and how it should evolve. Another set of potential cut-off points consists of *judicial decisions on maritime delimitation*, the consideration of which has been largely absent until recently (Yildiz and Yüksel 2024). By interpreting and applying customary and treaty rules, court and tribunals can create new law that may conflict with the preexisting set of legal rules. A judicial decision may interpret a rule in a way that is inconsistent with a treaty, with a previous ruling, or with what legal scholars or practitioners think to be the correct interpretation. Importantly, judicial decisions often specify what the customary law requires. The specific, written statements about what the custom is may clash with conflicting expressions found in treaties or the works of legal scholars.

In general, then, moments of *lawmaking* (multilateral or judicial) are especially susceptible of changing the coherence (or the degree of agreement) in law. Based on these considerations, I identify the following cut-off points:

1. The second UN Conference on the Law of the Sea (UNCLOS-II), which set out and failed to agree on an outer limit for the territorial sea, disclosing the stark diversity of state views on the issue in the process (April 26, 1960)
2. ICJ’s judgment in the *North Sea Continental Shelf* cases (February 20, 1969), which contradicted the 1958 Continental Shelf Convention on the primacy of equidistance as the delimitation method
3. Conclusion of the UNCLOS at the end of the third UN Conference on the Law of the Sea (UNCLOS-III) (December 10, 1982), which reduced the uncertainty over outer limits but not that over the delimitation of the continental shelf and the exclusive economic zone (EEZ)
4. ICJ’s judgment in the *Greenland/Jan Mayen* case (June 14, 1993), which represented a turn to a more predictable delimitation methodology that began with equidistance and adjusted it to achieve an equitable outcome.^16^

These cut-off and the degrees of uncertainty they delineate are summarized in Table 1.

**Table 1.** Baseline Legal Uncertainty in Maritime Boundary-Making.

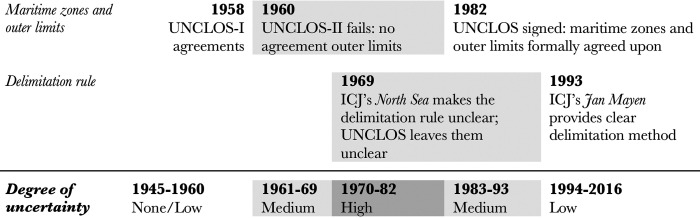

The first and the third cut-off points relate to the **extent of maritime zones**, which were subject to multilateral treaty making attempts. The first cut-off point is 1960, when UNCLOS-II ended without succeeding to set out an outer limit for the territorial sea, but only after revealing that the state practice on the matter was highly divided. A proposal of a maximum breadth of 6 nm fell one vote short of obtaining the required two-thirds majority (Friedheim 1965, 32-33).^17^ This failure followed the first UN Conference on the Law of the Sea in 1958 (UNCLOS-I), which had codified some of the emerging rules on state entitlement to a territorial sea and a continental shelf but had not set a maximum breadth for such zones. Legal scholars (including legal counsels working for states) debated whether the customary 3-nm limit still applied to the territorial sea, until another limit would be codified, or if, in light of state practice that moved beyond the 3-nm, the customary limit was already outdated and 6-nm and 12-nm claims were acceptable (Johnson 1961, 589-90). The third cut-off point represents the signature of the UNCLOS in 1982, by which time there remained no debate over the outer limits of the territorial sea or the other maritime zones such as the continental shelf and the EEZ.

The second and the fourth cut-off points relate to **maritime delimitation**. They represent two important instances when the ICJ was called upon to interpret the maritime delimitation rule with far-reaching effects. In 1969, with its *North Sea Continental Shelf* ruling, the ICJ stated that the equidistance principle—consisting of dividing the maritime zones between two states in the middle, with a line equally distant from both coasts—did not have a customary status or any privileged position among several possible delimitation methods. The uncertainty here was created because the ruling clearly contradicted the 1958 Continental Shelf Convention, which had all but raised equidistance to the status of a default rule, failing agreement on a different boundary line between states (Yildiz and Yüksel 2024). These conflicting interpretations on the place of the equidistance rule were durably brought into harmony only in 1993, which marks the fourth and the final cut-off point. In its 1993 *Jan Mayen* ruling, the ICJ rehabilitated equidistance as a first step to delimitation. This rule was further solidified by subsequent judicial rulings, and finally morphed into a three-stage methodology in the ICJ’s *Black Sea* ruling in 2009.^18^ Marking the point in the jurisprudence in favor of a more predictable rule, 1993 is an appropriate turning point around which uncertainty over the delimitation rule decreases. The importance of these two rulings have been noted by several legal scholars, who clearly point to how 1969 made things more complicated and 1993 brought in more predictability (Lando 2019; Tanaka 2019). Other judicial rulings and arbitral awards before 1993 tended to follow the lead of the ICJ’s 1969 ruling, while those after 1993 followed the ICJ’s lead in *Jan Mayen*,^19^ which further justifies these cut-off points.

The various cut-off points lead to three different degrees of uncertainty: *Low*, *Medium*, and *High* (see Table 1). The variable for baseline legal uncertainty is thus an ordinal one taking these three values.

#### Dyad-Specific Legal Uncertainty

In addition to this baseline uncertainty, some dyads may face additional uncertainty due to disagreements related to the effect of certain coastal or maritime features on the delimitation of maritime boundaries. Conversely, some dyads may be less susceptible to the effects of uncertainty due to the relative simplicity of their coastlines. Among possible factors that should be taken into consideration are (a) whether states involved have opposite or adjacent coasts to each other, (b) whether there are offshore islands in the area of delimitation, (c) and whether the delimitation area goes beyond the territorial sea. I discuss these three factors and their operationalization in turn.

Delimitation principles concerning states with *adjacent* coasts (typically, states with land contiguity with a land border reaching the coast) have been harder to establish, partly because choices made at the meeting point of the land borders are extremely consequential on the whole course of the boundary and harder to agree on. Conversely, equidistance appeared as a reasonable way to at least begin the delimitation in uniquely *opposite* coasts. The ICJ was ready to state in 1993 that the median line in the case of opposite coasts would be an equitable way of dividing up the maritime areas in principle, while it had to wait until 2001 (in its *Qatar v. Bahrain* decision) to make a similar statement for states with adjacent coasts (Evans 2015, 258-59). I create a dummy variable to capture whether the dyads have at least some adjacent coasts rather than exclusively opposite coast to delimit.

The existence of *offshore islands* controlled by one state but close to the coast of another state may provide a second factor of dyad-specific uncertainty. The question of how much weight such islands should have in maritime delimitation has been highly controversial, and courts and tribunals have not been uniform as they treated this matter.^20^ The question boils down to whether the islands should be given the same consideration in the drawing of a delimitation line as the mainlands, with states—depending on whether they own the islands in question or not—differing on their preferred answers. I create a variable that takes the value of 1 when there are *offshore islands* in the vicinity of the delimitation area whose effect may be subject to disagreement.^21^

The third factor that can pose a challenge for some dyads is *delimitation beyond the territorial sea*. The delimitation rules for territorial sea and broader zones^22^ can be said to have diverged in 1969 as the ICJ pushed aside equidistance as a preferred method beyond the territorial sea in its *North Sea* judgment. For the territorial sea, the delimitation rule had been based on equidistance since the 1958 Convention on the Territorial Sea (TSC) (art. 12), which was reaffirmed in the UNCLOS (art. 15). For continental shelf and EEZ delimitation, the UNCLOS only contained vague delimitation provisions that did not mention equidistance but rather pointed towards the need to reach an equitable solution (articles 74 and 83). As a consequence, uncertainty should affect states that have continental shelves or EEZs to delimit more than those that only have a territorial sea to delimit. I capture this with a dummy variable that takes the value of 1 when the areas to delimit go beyond the territorial sea and 0 otherwise.

### Control Variables

I consider a series of economic control variables that concern factors that increase the value of the maritime area for states and may incentivize extensive claims. Commentators have long argued that the existence of *resources* in maritime areas is a major driver of maritime boundary activity (see, e.g., the separate opinion of Judge Jessup in *North Sea*). In particular, offshore oil and gas (*hydrocarbon* resources) may increase the value of the delimitation area for states, leading them to make an extensive claim to control as much of the resource-rich area as possible. Similar considerations may hold for fisheries. Some of the first extensive claims were made by states that sought to limit foreign fishing in their waters (e.g., Peru and Chile’s 200-nm claims in the late 1940s) (Loring 1971).

The variable for hydrocarbon activity is derived from the Petroleum Dataset, which codes the years and locations of hydrocarbon discovery and production activities (Lujala et al. 2007). I overlay the discovery and production locations on a map of EEZ boundaries (Flanders Marine Institute 2023) to identify the dyads that may be concerned by the activity in question (see Codebook, OS Section S2). A dichotomous *hydrocarbon activity* variable takes the value of 1 from that year onwards. To capture the time preceding discovery, which can also make boundaries salient for states—the *exploration* period—I create a binary variable coded as 1 in the 3 years preceding discovery. As for fisheries, I use data from the Food and Agricultural Organization and calculate the *total catch* for each dyad-year. I take the logarithm of this variable due to its right-skewed distribution.

I also control for the existence of a *related territorial dispute* and a *maritime boundary dispute*. Disputes over territory may be important drivers of maritime disputes, especially if they are over areas that can affect how a maritime boundary may eventually be drawn. A dispute where two states’ land boundaries meet their coasts may lead to overlapping maritime boundary claims, the reconciliation of which may require the prior resolution of the territorial dispute. As Mitchell (2020) argues, states may also end up in maritime disputes as they pursue their territorial disputes. The information on territorial disputes has been collected using various available datasets (Hensel et al. 2008; Huth and Allee 2002; Schultz 2017). This variable takes the value of 1 from the year in which a territorial dispute relevant to the plausible location of the maritime boundary begins. I similarly include a dichotomous variable that takes the value of 1 from the moment in which there exists a maritime boundary dispute. While territorial disputes may make states more likely to enter into maritime boundary disputes, existing maritime boundary disputes should reduce the likelihood of similar disputes in the same dyad.^23^ Finally, two variables concern national capabilities and regime type. To create a measure of asymmetry in capabilities, I use the Correlates of War (COW) dataset including composite national capabilities (Singer et al. 1972). I take the ratio of the capability score of the stronger state to the dyadic total. Regarding the regime type, I create a variable that captures whether the dyad is jointly democratic, includes one democracy and one autocracy, or is jointly autocratic. I use the typical cut-off of 6 points on the Polity IV scale (Marshall and Gurr 2020), with states scoring 6 or more considered to be democracies. Except for hydrocarbon exploration (lagged by design), capability ratio, and regime type, I lag all variables by 1 year.

### Estimation Strategy

I run fixed-effects logistic regressions, modelling the logged odds of dispute onset as a function of either baseline or dyad-specific uncertainty and a series of predictors. Baseline uncertainty is dictated by variations across time that affect dyads at specific periods. I use dyad-fixed effects to get at how a dyad’s dispute probability evolves as it is subjected to different levels of baseline uncertainty over time. The regression equation is:

(1) $$log\left(\frac{p_{it}}{1-p_{it}}\right)=\beta X_{it}+\alpha_{i}+\varepsilon_{it}$$

where *p*_*it*_ is the probability of dispute onset for a given dyad *i* and year *t*, **X** is the vector of predictors and ***β*** is the vector of coefficients associated with them, *α*_*i*_ represent the dyad-fixed effects, and *ɛ*_*it*_ is the error term. In these models, I omit variables that do not change (or change very little) over time.^24^

Another set of tests focus on the effect of dyad-specific factors. In these, I control for **common shocks** by using year-fixed effects. The equation is as follows, where *γ*_*i*_ represents year fixed effects:

(2) $$log\left(\frac{p_{it}}{1-p_{it}}\right)=\beta X_{it}+\gamma_{i}+\varepsilon_{it}$$

In both sets of models, I control for time dependency by adding duration as a cubic polynomial, as suggested in Carter and Signorino (2010). The duration, *t*, captures the time from the beginning of the observation period, the year of independence in case of entry at a later date, or the year in which the dyad experienced its last maritime boundary dispute onset. In practice, I include *t*, *t*^2^, and *t*^3^ as regressors.

## Analysis

**Figure 1.**
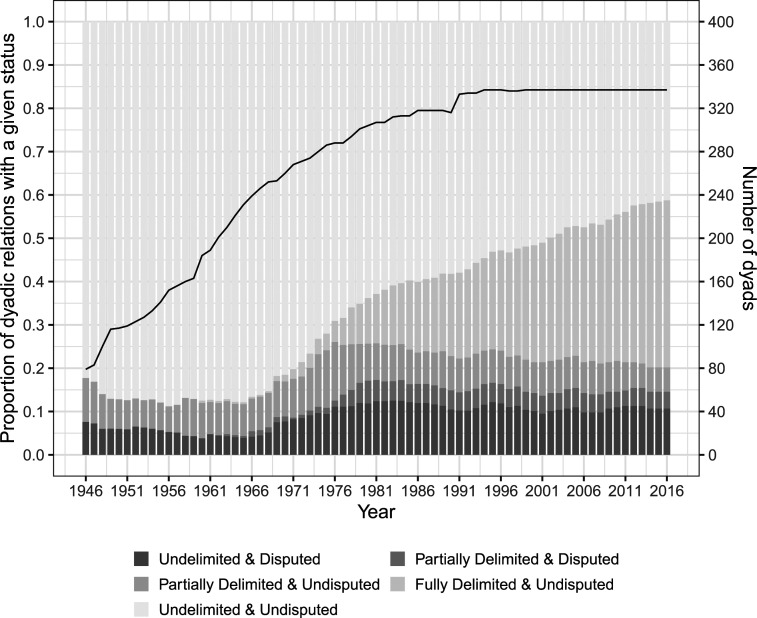
Distribution of dyad-years according to boundary status over time.

Figure 1 presents the evolution of dyadic maritime boundaries in the observation period in terms of their delimitation and dispute status. Across the observation period, most dyads have had *undelimited* and *undisputed* boundaries. From the mid-1970s, a sizeable group of dyads managed to successfully and fully delimit their common maritime boundaries. As Figure 1 suggests, dyads that experience maritime boundary disputes, represented by the two darker shades of gray, are just a small subset of dyads that have common maritime boundaries to delimit.

A dispute onset is observed in only 103 dyads between 1946 and 2016, and in about 90 percent of them, only once.^25^ In total, there are 116 unique onsets. A new dispute begins in less than 1 percent (about 0.6 percent) of the 18,999 dyad-year observations. This proportion is barely superior when we consider only those dyad-years with risk of dispute onset (about 0.7 percent of the 15,776 dyad-year observations). Due to the rareness of the outcome of interest, I run additional tests with penalized maximum likelihood, following Cook et al. (2020).

### Baseline Legal Uncertainty and Dispute Onset

**Figure 2.**
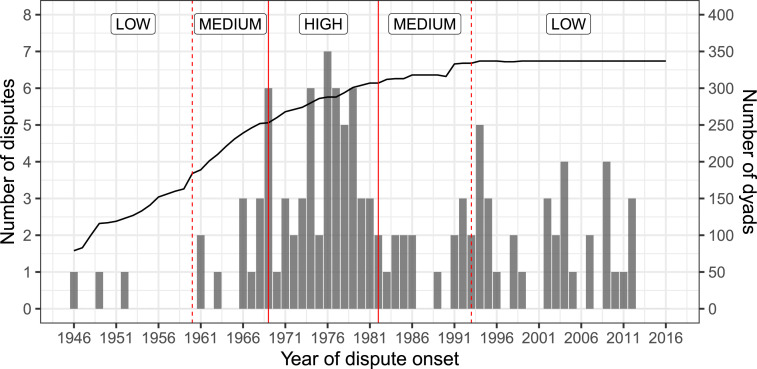
Number of new disputes for each year under study. The number of dyads is indicated by the solid black line corresponding to the second *y*-axis. Vertical lines divide periods of different baseline uncertainty levels.

To assess the face validity of the hypothesized positive relationship between high legal uncertainty and probability of dispute onset, Figure 2 displays the number of new disputes over the years under study with periods constructed based on the different levels of baseline legal uncertainty. This first look suggests that more disputes begin when legal uncertainty is high compared to other periods. Yet, patterns do not fit neatly within the periods. For instance, the period after 1993 corresponding to a lower level of uncertainty sees dispute onset in numbers comparable to the period with medium level of uncertainty.

I further study this possible relationship with a battery of tests. First, I run logistic regressions with dyad-fixed effects, modelling the probability of dispute onset as a function of baseline legal uncertainty. The regression output is presented in Table 2. Models 1 and 2 focus on baseline uncertainty, Models 3 and 4 also include controls. Models 2 and 4 use penalized maximum likelihood, as suggested in Cook et al. (2020). The estimates of legal uncertainty are on the whole consistent with the possible role of legal uncertainty in driving new disputes.^26^

**Table 2.** The Relationship Between Baseline Legal Uncertainty and the Probability of Dispute Onset.

	FE Logit	Penalized ML	FE Logit	Penalized ML
MEDIUM legal uncertainty (ref: LOW)	1.72**	1.55***	2.37^+^	1.69***
	(0.55)	(0.22)	(1.32)	(0.24)
HIGH legal uncertainty (ref: LOW)	2.73***	2.49***	3.25*	2.53***
	(0.64)	(0.26)	(1.34)	(0.32)
No. of dyadic boundaries to delimit (lagged & logged)			0.61	0.02
			(1.53)	(0.61)
Joint UNCLOS (ref: Before UNCLOS)			−1.32	−1.15
			(1.97)	(0.40)
Neither UNCLOS (ref: Before UNCLOS)			−0.18	−0.05
			(0.68)	(0.21)
One state UNCLOS (ref: Before UNCLOS)			0.42	−0.07
			(1.22)	(0.26)
Hydrocarbon exploration			1.74**	1.34***
			(0.60)	(0.22)
Hydrocarbon activity			0.63	0.49
			(0.62)	(0.33)
Ongoing maritime boundary dispute (lagged)			−3.03**	−2.63***
			(0.94)	(0.43)
Ongoing related territorial dispute (lagged)			1.47	1.14*
			(1.00)	(0.37)
One side DEMOCRATIC (ref: joint AUTOCRACY)			0.34	0.47
			(1.09)	(0.30)
Joint DEMOCRACY (ref: joint AUTOCRACY)			0.50	0.64
			(0.86)	(0.28)
Dyad-fixed effects	Yes	Yes	Yes	Yes
Year-fixed effects	No	No	No	No
Cubic polynomial for time	Yes	Yes	Yes	Yes
Num. obs.	4,672	4,672	2,229	2,229
Num. groups: dyad	103	103	63	63

***p < .001; **p < .01; *p < .05; ^+^*p* < .1.

Robust standard errors clustered by dyad in parentheses.

**Figure 3.**
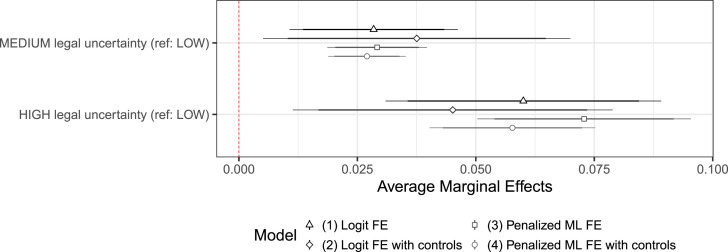
Average marginal effects of baseline uncertainty on the probability of dispute onset.

Next, I calculate average marginal effects based on these four models, present them for baseline uncertainty in Figure 3 and the rest in Figure S4.1 (OS). In each model, the average marginal effect of higher levels of uncertainty is positive and substantively large. Compared to when baseline legal uncertainty is low, medium levels of uncertainty are associated with an increase of around 3 percentage points in the probability of dispute onset, and high levels of uncertainty correspond to effects that hover around 5–6 percentage points. This increase in probability is comparable to the decrease in probability of a new dispute onset for dyads that are already involved in a dispute. Only hydrocarbon exploration and subsequent activity have a comparable positive association with the probability of dispute onset. Given that these effects are obtained by removing the effects of time-invariant confounders, we can interpret them as an increase in the likelihood of dispute onset for states as they go through periods of higher legal uncertainty.

Fixed-effects models have the advantage of focusing on changes within units, thus making it possible to assess how the probability of dispute evolves as the surrounding uncertainty evolves. Yet, it is not possible to include time-invariant variables. As such, these models do not allow us to test our expectations about the relationship between the dyad-specific components of uncertainty (based on geographic factors that rarely change over time) and dispute onset.

### Dyad-Specific Uncertainty and Dispute Onset

To get to the effect of dyad-specific uncertainty, I run logistic regressions with year-fixed effects that remove the effect of common shocks across time. Adding year-fixed effects essentially removes the baseline component of uncertainty. The regression output is presented in Table 3. Offshore islands and adjacency of coasts have estimated coefficients that are different from zero at conventional levels, while this is not the case for the third dyad-specific component. Other control variables do not behave surprisingly, the only new result here being that compared to joint autocracies, democracy-autocracy pairs are more likely to experience onset.

**Table 3.** The Relationship Between Dyadic Legal Uncertainty Factors and Dispute Onset.

	FE Logit	Penalized ML	FE Logit	Penalized ML
Offshore island	0.92***	0.92***	0.98***	0.96***
	(0.19)	(0.18)	(0.29)	(0.25)
Adjacency of coasts	0.69***	0.69***	0.59*	0.58*
	(0.20)	(0.14)	(0.28)	(0.23)
Beyond territorial sea	0.34	0.28	1.16	0.82
	(0.47)	(0.29)	(1.03)	(0.35)
No. of dyadic boundaries to delimit (lagged & logged)			−0.31	−0.31
			(0.28)	(0.18)
Hydrocarbon exploration			1.44***	1.40***
			(0.37)	(0.29)
Hydrocarbon activity			0.12	0.13
			(0.27)	(0.17)
Total catch (lagged & logged)			0.16^+^	0.16*
			(0.09)	(0.06)
Capability ratio			−1.72*	−1.70*
			(0.81)	(0.56)
Ongoing maritime boundary dispute (lagged)			−1.32**	−1.26***
			(0.49)	(0.30)
Ongoing related territorial dispute (lagged)			0.95***	0.93***
			(0.28)	(0.24)
One side DEMOCRATIC (ref: joint AUTOCRACY)			0.99**	0.98***
			(0.35)	(0.27)
Joint DEMOCRACY (ref: joint AUTOCRACY)			0.39	0.38
			(0.29)	(0.20)
Dyad-fixed effects	No	No	No	No
Year-fixed effects	Yes	Yes	Yes	Yes
Cubic polynomial for time	Yes	Yes	Yes	Yes
Num. obs.	10,665	10,665	4,516	4,516
Num. groups: year	44	44	28	28

***p < .001; **p < .01; *p < .05; ^+^*p* < .1.

Robust standard errors clustered by year in parentheses.

**Figure 4.**
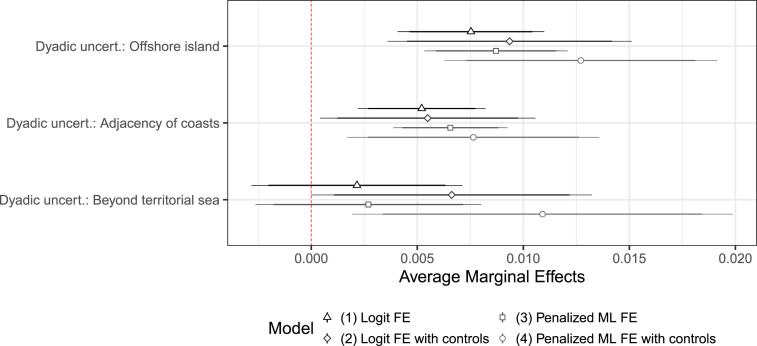
Average marginal effects of dyadic uncertainty on the probability of dispute onset.

I calculate marginal effects based on these models and present them in Figure 4 for the dyad-specific component of uncertainty and in Figure S4.2 (OS) for all variables.

Controlling for common shocks associated with time, including baseline legal uncertainty, each of the dyad-specific component appears to be positively associated with the probability of dispute onset. The association is the strongest in the case of offshore islands. Dyads that have offshore islands in the delimitation area can expect to see their risk of entering into a dispute increase by almost one percentage point. Adjacency of coasts comes next. Having areas to delimit beyond the territorial sea makes a positive contribution as well, although the computed marginal effects are not distinguishable from zero in most model specifications.

### Robustness Checks

While the two sets of tests above attest to the plausibility of the link between legal uncertainty and dispute onset, the findings can be driven by alternative explanations. I carry out a series of robustness checks to assess this.

First, I check whether disputes appear more likely to begin during times of high legal uncertainty because the period overlaps with other developments making maritime boundary-making salient for states. During episodes of multilateral negotiations on the law of the sea, many states may feel compelled to stake a claim and some of their positions may conflict with one another. Including a variable that is coded as 1 during the negotiation periods of UNCLOS-I (1958), UNCLOS-II (1960), and UNCLOS-III (1973–1982) does not change the association between variables related to legal uncertainty (especially baseline legal uncertainty) and dispute onset. Second, I test three period variables that may rival baseline legal uncertainty in explaining some of the temporal variation in patterns of dispute onset. In two separate models, I include variables to stand for the post-Cold War period on the one hand, and the period after the signature of the UNCLOS and that after its entry into force on the other hand. The results are reported in Table S5.1 (OS), with average marginal effects plotted in Figures S5.1 and S5.2 (OS). No evidence of statistically significant relationship is found for these alternative period variables, except for the entry into force of the UNCLOS. Throughout these tests, the relationships detected with respect to legal uncertainty variables remain robust on the whole, except in one specification where the estimates fall short of conventional statistical significance levels.

A second set of robustness checks primarily addresses dyad-specific uncertainty. To begin with, I include variables for domestic legal system (Powell and Mitchell 2007) and recent independence. The results are reported in Table S5.2 and Figure S5.3 (OS). Again, the results stand. Then, I test whether dyad-specific uncertainty seems particularly more likely to drive disputes in times when baseline legal uncertainty is also high through a dynamic difference-in-differences approach. For this, I consider treatment to be any dyad-specific uncertainty factor and look for effects post-1969 (for details, see OS, Section S5.3). For dyads with dyad-specific uncertainty, dispute becomes more likely after 1969, when baseline uncertainty is high, and largely goes back to its pre-1969 levels when baseline uncertainty decreases. This suggests that dyad-specific factors successfully get at drivers of disputes both on their own and in combination with baseline uncertainty.

Third, I consider regional patterns. While there are differences across regions, the results about baseline uncertainty and the two aspects of dyad-specific uncertainty (offshore islands and adjacency) are consistent with results obtained without distinction of region. Section S5.4 of the OS further comments on these patterns.

Fourth, I run a test that considers that the time before 1958 is not one of low legal uncertainty but that of legal void. The inclusion of legal void (which takes the value of 1 in years before and including 1958) does not affect the relationship between legal uncertainty and dispute onset (see OS, Section S5.5). Finally, I run Poisson and negative binomial regressions to assess the relationship between the annual baseline legal uncertainty score and the number of new disputes in a given year. Results confirm the dispute-generating potential of high legal uncertainty (see OS S5.6).

### Probing the Mechanism

The mechanism I propose suggests the following path: legal uncertainty increases the probability that parties make incompatible claims based on a selective and possibly maximalist reading of alternative rules and interpretations that favor them the most. As alternative rules and interpretations proliferate, this path is likely to be activated in more dyads, and therefore, dispute onset is more likely. In this section, I probe the mechanism quantitatively and illustrate it using a case study.

As a first, quantitative approximation, I measure the compatibility of claims on paper and assess whether the degree of *match* between two states’ positions is related to legal uncertainty and dispute onset in the way the theory predicts. I code the degree of match between states’ positions in two dimensions. The first considers if two states make the same numerical outer limit claim for their maritime zones. For instance, if both claim 12 nm for their territorial sea limits and 200 nm for their continental shelves and EEZs, there is a match. The second dimension considers whether states support the same delimitation method. I use data on state preferences over delimitation methods from Yildiz and Yüksel (2024), recording whether states prefer equidistance, modified equidistance, or non-equidistance. While these are records of states’ delimitation method preferences over the continental shelf, they can be extended to the EEZ since within 200-nm these two zones are practically delimited in the same way. The provisions of the UNCLOS concerning the EEZ (art. 74) and CS (art. 83) delimitation are identical, and there is common state practice of drawing a single maritime boundary that covers both the CS and EEZ (Oxman 1993, 35).

I first check whether baseline uncertainty makes a match less likely. I do this for the territorial sea (TS), fishing zone/EEZ (FZ/EEZ), and continental shelf (CS) separately. I also look for matches in the CS delimitation method (CS DLM). The regression output is presented in Table 4. The results suggest that the probability of having matches has a negative association with legal uncertainty—high levels of legal uncertainty correspond to a lower probability of matching (see OS S6.1 and S6.2).

**Table 4.** The Relationship Between Baseline Uncertainty and Matches. TS: Territorial Sea; FZ/EEZ - Fishing Zone/Exclusive Economic Zone; CS: Continental Shelf; DLM: Delimitation Method.

	TS Match	FZ/EEZ Match	CS Match	CS DLM Match
MEDIUM legal uncertainty (ref: LOW)	−0.66*	−1.52***	−2.25***	−0.67***
	(0.23)	(0.23)	(0.26)	(0.13)
HIGH legal uncertainty (ref: LOW)	−1.66***	−2.03***	−2.69***	−1.41***
	(0.25)	(0.22)	(0.28)	(0.14)
Dyad-fixed effects	Yes	Yes	Yes	Yes
Year-fixed effects	No	No	No	No
Cubic polynomial for time	No	No	No	No
Num. obs.	866	1,044	806	2,422
Num. groups: dyad	206	220	153	252

***p < .001; **p < .01; *p < .05; ^+^*p* < .1.

Robust standard errors clustered by dyad in parentheses.

**Figure 5.**
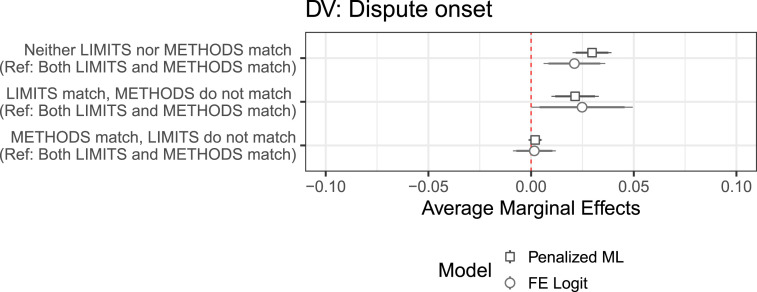
Average marginal effects of match type on dispute onset.

While these tests look at limits and delimitation methods separately, I consider which type of mismatch may be driving new disputes in another set of tests. I create a categorical variable that can take four values: both the limits and the methods match (when both TS and EEZ limits match),^27^ only the limits match, only the methods match, and neither the limits nor the methods match. Figure 5 shows marginal effects calculated in models with dispute onset as the outcome (see OS S6.1 for the regression output).

This reveals that disputes are not more likely to arise because states make* different outer limit claims*. Instead, *different delimitation methods* appear to make disputes more likely, both when limits match and when they do not. Outer limit claims differ as one state extends its jurisdiction in the sea—say from 3 to 12 nm in the territorial sea—or claim a new zone—for example, a 200-nm EEZ—before its neighbor. Usually, what the neighbor then does is to match the claim, instead of calling into question the state’s right to extend its jurisdiction. Most disputes in the data result from the disagreement about which delimitation method to use, as states pick and choose the method among available alternatives that allows them to justify asking for a greater area. To be sure, especially in times of high legal uncertainty impacting both outer limits and delimitation methods, more disputes over outer limits could have arisen if some states had made extensive outer limits claims just for themselves while denying their neighbor’s right to do the same. Instead, as noted in the theory section, states’ initial claims appear to be limited by what they are willing to concede to their neighbors—one can claim a 200-nm EEZ, but not without admitting that its neighbor can do the same. This suggests that law not only pushes for extensive claims but also constrains claims, notably due to the principle of equality between states. Thus, the general extension of limits in the sea seems to contribute to dispute onset only by increasing the area that is to be delimited, at which point the uncertainty over the delimitation method takes center stage in driving disputes.

The Aegean dispute between Greece and Turkey, which began in 1974, provides another illustration of the importance of legal uncertainty, especially that concerning the delimitation method. Contemporary commentators attributed its origins to the negotiations during UNCLOS-III, suggesting that these negotiations made the issue salient for both states (Buzan 1978; Wilson 1979). Yet the salience of the issue on its own does not explain how states came to adopt incompatible legal claims and entered into a dispute.

Throughout the 1960s, both Greece and Turkey favored the same rule of delimitation as enunciated in the 1958 CSC whereby equidistance was the default rule unless parties agreed otherwise. In April 1959, Greece made a continental shelf claim (Act No. 3948 of 17 April 1959 Concerning the Exploration and Exploitation of Hydrocarbons), based on which it carried out a series of exploration and exploitation activities in the 1960s, without any Turkish protest (Ergüven 1982, 152-3; Gürel 2018, 143). While Turkish legislation at the time did not talk of a continental shelf, its 1964 legislation provided that the delimitation of the territorial sea would be carried out on the basis of the equidistance principle, and that islands would have the exact same territorial seas as the mainland (Law on the Territorial Sea, articles 3 and 6). This was in line with what the 1958 TSC provided for the delimitation of the territorial sea, almost the same as what the 1958 CSC provided for the continental shelf.

The dispute only took shape as the primacy of equidistance as a delimitation rule was put into question by the ICJ in its 1969 *North Sea* decision, where the court rejected the customary status of equidistance as the default rule and proposed a different delimitation method based on *equitable principles*. Moreover, it highlighted the notion of *natural prolongation*. This ruling provided Turkey an opportunity to argue against the application of equidistance in the Aegean Sea, push for the use of equitable principles and argue that some of the Aegean islands lying close to Turkey laid on Turkey’s natural prolongation.

One 7 February 1974, Greece protested Turkish exploration licenses issued a few months earlier on the basis that areas covered by Turkish concessions overlapped to a great extent with the continental shelf that would be generated by the neighboring Greek islands. The protest laid out Greece’s view that islands were entitled to a continental shelf just like mainland territories, and an equidistant line should be drawn between the Greek islands and the Turkish coast (Rozakis 1997, 94).^28^ In response, Turkey rejected the equidistance principle, making direct reference to the ICJ’s 1969 *North Sea* judgment. Since then, Turkey insisted on the inapplicability of the equidistance principle in the Aegean Sea, and argued that delimitation should be guided by the notions of natural prolongation and equity. It continued to invoke these arguments in further diplomatic notes as well as during UNCLOS-III. Greece, for its part, insisted on the application of the 1958 CSC, which inscribed the equidistance principle as a default rule and held that islands had full entitlements to maritime zones (Gross 1977, 32-34).

### Limitations

An important limitation concerns the difficulty of testing the mechanism more precisely. There are at least two challenges. The first relates to our ability to detect compatible and conflicting unilateral claims. Even with data on unilateral claims, our measurements may not be granular enough to capture the nature and extent of the incompatibility between the claims made. Datasets are not detailed enough to pin down how exactly states are drawing their baselines and how they treat islands, rocks, or other features as they make their claims. Moreover, two states may appear to make the same numerical claims on paper, but may still enter into a dispute because they insist on the full extent of their claim and demand that the other contend itself with less. A second reason is the question of perceptions. Claims that appear limited may be read by one or the other side as excessive, because they may be considered as an attempt to impose a boundary instead of staking an initial negotiation position. The conflict between claims is thus not easy to capture just by looking at unilateral claims as they can be realistically measured and compared across dyads over time.

Another limitation is the difficulty of pinpointing the effect of legal uncertainty on shaping the precise timing of a dispute. Tests made in different regions reveal that maritime boundary activity varies in intensity not only over time but also across space. Disputes are seemingly shaped not only by legal uncertainty present at the time, but also other factors that push states to make initial claims and negotiate over them. Thus, the period when legal uncertainty is the highest may not be the one most conducive to disputes globally. Yet, when an interest materializes—for instance, as states in the same region begin to delimit their boundaries or resources are found—the law can still be used to express different claims, and the degree of uncertainty still matters. Future studies can usefully assess when and how legal uncertainty drives disputes in combination with other interests states may have in making a claim at a specific point in time.

## Conclusion

Understanding the state of the international law can help us make sense of the variation in how neighboring states have dealt with their maritime boundaries in the aftermath of World War II. Legal uncertainty appears to be positively associated with the probability of dispute onset, likely because states are interested in controlling greater areas and can find in uncertain law alternative rules and interpretations to maximize their claims. High levels of legal uncertainty would thus increase the risk that states make maximalist claims that would overlap with each other. The analysis, carried out on a novel dataset, corroborates this expectation.

An important area for future research can be about establishing the precise mechanisms by which law can shape unilateral claims, dispute origins and dispute outcomes. While I suggest that the relationship is plausibly sustained by the tendency of uncertain law to invite maximalist claims, further in-depth studies can usefully add in more direct evidence through archival material and possibly interviews with policymakers and legal counsels involved in shaping states’ maritime boundary policies. Moreover, experimental evidence can be helpful in establishing the psychological mechanisms by which an adversary’s claim appears maximalist and how international law interferes in the process.

An important contribution of this study lies in the way it problematizes the role of legal rules in dispute processes. The success of new legal rules in providing more certainty and higher likelihood of peaceful dispute management should not be taken for granted but tested empirically. At the same time, the existence of contention should not be construed to mean that laws are failing. That lawmaking in the sea created new disputes between some actors does not necessarily reflect badly on legal rules, especially if such disagreements remain within the legal realm instead of spilling over to militarized conflict. Future studies should consider whether disagreements with legal origins are managed in ways that differ from disagreements primarily tied to territory, resources, or rivalry dynamics.

Beyond dispute onset, the possible impact of legal uncertainty on dispute management can usefully be fleshed out, especially in issue areas with a certain degree of presence for international law, such as international trade, investment, and armed conflict. Future work can also make the concept of legal uncertainty more useful by theorizing and measuring it in a more disaggregated manner. It may, for instance, consider the number of and the distance (in meaning and consequences) between contending rules and interpretations. We may expect legal uncertainty to operate in a certain way when there are just two contending rules, clearly different in their effects; and the expectations should differ if we instead face dozens of alternative interpretations that differ only slightly in their implications.

More generally, future work could usefully consider in a more fine-grained manner how the various political, economic, and strategic interests of states interact with the incentives, opportunities, and constraints provided by laws, institutions, and domestic politics. Comparative and case-based studies could be useful to test the mechanisms by which states respond to systemic changes, while contending with domestic constituencies and pursuing other goals in line with their interests. Although developed in the domain of maritime boundary relations, the theory I propose has implications for other issue areas where both distributional and zero-sum questions arise, implying both maximalist tendencies and interest in cooperation for joint gains.

## Supplemental Material

Supplemental Material - The Law Behind Dispute Onset: How Legal Uncertainty Drives Maritime Boundary Dispute Supplemental Material for The Law Behind Dispute Onset: How Legal Uncertainty Drives Maritime Boundary Dispute by Umut Yüksel in Journal of Conflict Resolution

Supplemental Material - The Law Behind Dispute Onset: How Legal Uncertainty Drives Maritime Boundary Dispute Supplemental Material for The Law Behind Dispute Onset: How Legal Uncertainty Drives Maritime Boundary Dispute by Umut Yüksel in Journal of Conflict Resolution

## Data Availability

The datasets generated and analyzed during this study as well as replication code are available at Harvard Dataverse (Yüksel 2024): https://dataverse.harvard.edu/dataset.xhtml?persistentId=doi%3A10.7910%2FDVN%2FOSKGUR.

## Notes

1 I use claim to designate unilateral claims, and disputes to stand for competing claims made by two or more parties. This usage differs from scholarship that uses claims to stand for competing claims, and disputes to designate militarized conict. I make this distinction because most unilateral boundary claims do not become an interstate dispute, as, unlike in territorial disputes, a claim may be made to an area that belongs to (or is controlled by) no one.

2 See https://www.paulhensel.org/icowmar.html.

3 Coastal states include any state with a coastline bordering on open seas, thus excluding landlocked states as well as those that abut waters not giving access to another sea or the ocean (i.e., the Aral Sea, the Dead Sea, and the Caspian Sea).

4 If other states consider one’s claims excessive, they may ignore or actively contest them by continuing to carry out fishing, resource exploration and exploitation, and various military activities in the claimed areas. If the claimant state then wants to impose its will, it has to bear the cost of enforcing its jurisdiction in some from, for instance, by the use of its coast guard, and run the risk of potential conflict. These costs and risks may in effect cancel out all the benefit that a state wanted to obtain by establishing exclusive jurisdiction in the first place.

5 This also applies to the nature of rights claimed within a maritime area—if a state claims exclusive jurisdiction over resource exploration and exploitation in a given maritime area, it must also admit that other states would have the same right in their respective maritime areas.

6 As Verdier and Voeten (2015) point out, claims made by virtue of customary international law by one state implies that state’s recognition of the right of other states to make similar claims.

7 Maritime issues examined in the ICOW project comprise a combination of maritime boundary disputes and challenges to maritime jurisdiction. In the first case, we are dealing with a disagreement over how to delimit a common maritime boundary. In the second, what is at stake is how a state responds to a claim made by another state when there are no prospects of common maritime boundaries (for instance, when the US challenges Peru’s extended fishery claims). The data and analysis in this paper address the first set of cases—those that are about where a common boundary should be drawn. I make no claim about when broader disagreements over maritime jurisdiction may arise.

8 See https://brill.com/view/serial/IMBO.

9 See https://www.un.org/depts/los/doalos_publications/los_bult.htm.

10 See https://www.un.org/depts/los/LEGISLATIONANDTREATIES/regionslist.htm.

11 For the latest edition, see https://www.jag.navy.mil/organization/code_10_mcrm.htm.

12 See https://www.state.gov/limits-in-the-seas/.

13 For news related to boundaries, see https://www.durham.ac.uk/research/institutes-and-centres/ibru-borders-research/news-and-events/boundary-news/. For other relevant publications such as maritime briefings, see https://www.durham.ac.uk/research/institutes-and-centres/ibru-borders-research/maps-and-publications/publications/.

14 See https://www.cia.gov/the-world-factbook/countries/.

15 The provision reads: “2. A fundamental change of circumstances may not be invoked as a ground for terminating or withdrawing from a treaty: (a) if the treaty establishes a boundary; [⋯ ]”.

16 An earlier ICJ judgment (*Libya/Malta* in 1985) had already used this method while staying true to the principles laid out in 1969. The 1993 decisions represents a clearer turn towards systematically beginning with equidistance.

17 Lawmaking attempts do not need to succeed to contribute to the generation of uncertainty. A high-profile conference that fails can reveal the extent to which state views differ, with each contending view able to attract new adherents going forward.

18 It must be noted that a decision the ICJ made in 2007 did not begin with equidistance, but the fact that the 2009 ruling followed it so closely and that further rulings followed the three-stage methodology suggests that it was an exception rather than a real turn in jurisprudence. In any case, the 2007 ruling did not create a new alternative but instead used a nonequidistance-based method like in 1969.

19 With the exception of the 2007 ruling, see above.

20 Some systemization attempts have been made. For instance, in the second phase award of the Eritrea/Yemen arbitration, the tribunal applied what has been called an “integrity test,” whereby islands are given full effect (i.e., they can generate up to 12-nm of territorial sea and 200-nm of continental shelf and EEZ) if they form an integral part of the coastal configuration of the mainland. Other solutions used have been partial effect or no effect (enclaving) (Tanaka 2015, 215-17).

21 This question arises for rather distant islands that belong to one state and lie in the proximity of another state, or where islands may have a significant impact on where a delimitation line would be drawn. These belong to the group of “dependent islands” as referred to by Weil (1989, 52). Dyads involving island-states—such as Cyprus or Sri Lanka—are not coded here as involving offshore islands.

22 The continental shelf and the EEZ, reaching 200-nm. Equidistance was even more central when the question was that of delimiting the contiguous zone–now typically reaching 24-nm.

23 As a dyad’s common maritime boundary area is not unlimited, any existing dispute may already cover most if not all of the area that both states can plausibly claim, after which there may be no further areas to dispute.

24 This is the case for national capabilities and total catch.

25 Nine dyads had two disputes, only two had three, and none had more than three.

26 The intercepts and coefficients for cubic polynomial terms are omitted from all models.

27 I do not rely on declared continental shelf limits because there is a great variety of them and many are not entirely numerical, unlike the TS and EEZ limits.

28 The case is thus a good example of both the baseline uncertainty and the dyad-specific component of offshore islands, the so-called “distant island problem” (Karl 1977).
