# Supplementary material for: The Law Behind Dispute Onset: How Legal Uncertainty Drives Maritime Boundary Disputes
Source: J Conflict Resolut. 2024 Dec 5;69(7-8):1172–204. doi: 10.1177/00220027241305076 (PMC12274024; doi:10.1177/00220027241305076)
Supplement: Supplemental Material - The Law Behind Dispute Onset: How Legal Uncertainty Drives Maritime Boundary Dispute [file sj-pdf-1-jcr-10.1177_00220027241305076.pdf]

Online Supplement

The Law Behind Dispute Onset: How Legal Uncertainty Drives Maritime Boundary Disputes

Umut Yüksel*

29 November 2024

Contents

S1 Data set construction 1

S2 Codebook 3

S3 Descriptive tables and figures 6
- S3.1 The state of dyadic maritime boundaries over time 6
- S3.2 Summary statistics of the numerical variables used in models 6

S4 Marginal effects including controls 7

S5 Robustness checks 9
- S5.1 Alternative period variables 9
- S5.2 Legal origin and recent independence 11
- S5.3 The relation between dyadic and baseline uncertainty 13
- S5.4 Regional patterns of dispute onset and legal uncertainty 14
- S5.5 Legal uncertainty vs. legal void 17
- S5.6 Number of disputes and legal uncertainty 18

S6 Figures and tests probing the mechanism 19

*Department of Political and Social Sciences, Universitat Pompeu Fabra, Barcelona; Global Governance Centre, Geneva Graduate Institute, Geneva. **Email:** umut.yuksel@upf.edu, **ORCID**: 0000-0001-8561-3559.

S1 Data set construction

To have a maritime boundary dispute, two states must have common boundaries to delimit—they have to be **delimitation-relevant dyads**. I assess whether a dyad is delimitation-relevant in a given year on the basis of three considerations: (a) the states' *proximity* to each other, (b) the unilateral maritime boundary *claims* they have made, and (c) the state of *international law* regarding the limits of state jurisdiction in the sea.

Beginning with the *proximity* criterion, I first identify states that are delimitation-relevant *today.* These are land contiguous pairs of states except for those involving a land-locked country, and all states that are separated by less than 400 nautical miles (nm) of sea, given that the acceptable extent of state jurisdiction for most purposes is 200 nm today. To identify the second group, and to make sure that they have common maritime boundaries to delimit, I use the 200-nm EEZ boundaries mapped for each country in the maritime boundaries database provided by Marine Regions (Flanders Marine Institute 2023). After thus identifying the set of delimitiation-relevant dyads today, and considering that the acceptable limits of state jurisdiction did not always reach 200-nm historically, I determine *when* these delimitation-relevant dyads found themselves adjacent or close enough to each other to create the possibility that they would need to delimit a common maritime boundary. For states that have a land border extending to a shared coastline, I judge that the possibility of observing a dispute begins at the same time as land contiguity. For states that do not share this type of land contiguity, the possibility of overlapping claims arises in one of the following ways.

First, a unilateral state act may create a risk of overlap, which can materialize if the neighboring state makes a reciprocal claim. This can either occur through a state making a claim to extended jurisdiction or becoming party to a treaty that specifically allows it to do so. Relevance may begin when one of the states makes a claim which, if reciprocated by a neighboring state, can lead to an overlapping claim. For instance, if a state makes a 50-nm claim, dyads including that state and other states that are separated by less than 100-nm of waters become relevant. If the claim is of unspecified breadth—which was quite common in early continental shelf claims—I take it to reach 200 nm, as this was the extent to which a few states declared continental shelves from the late 1940s. For instance, relevance between Cuba and Honduras, separated by about 300-nm of waters, was coded to begin in 1954, the year in which Cuba made a unilateral continental shelf claim of unspecified extent. Similarly, the risk of onset may begin by a state's ratification of, or accession to a treaty that allows it to extend its jurisdiction in the sea—such as the 1958 Continental Shelf Convention (CSC) or the 1982 United Nations Convention on the Law of the Sea (UNCLOS). For example, relevance between Albania and Italy begins in 1964 when Albania acceded to the CSC.

Second, a clear change in the international legal context towards broader outer limits creates a risk for states that find themselves closer than twice those limits. Here one must especially consider changes in the customary international law that can entitle all states—

regardless of treaty ratification—to extended state jurisdiction. Accordingly, relevance for all the states separated by less than 400 nm was coded to begin from 1975 at the latest, the year on which states negotiating the new law of the sea treaty during UNCLOS-III agreed on the maximum limit of 200-nm for the EEZ (see the Informal Single Negotiating Text (1975), part of the official records of UNCLOS-III, and which can be accessed at http://legal.un.org/diplomaticconferences/1973_los/vol4.shtml under *Working Papers of the Plenary*).

Based on these criteria, the dataset identifies 444 dyads that were relevant for delimitation at some point in time. The total number of dyads that are delimitation relevant at a given year ranges between 79 (in 1946) and 337 (from 1994 onwards). Table S3.1 in the next section of this Online Supplement (OS) provides the number of dyads and the status of their maritime boundaries at ten-year intervals. A detailed set of dyads involved as well as justifications as to when they were considered to be delimitation-relevant and whether the dyad was related to others (due to decolonization or state succession, for instance) can be provided upon request.

A caveat is in order. There are more dyads that may be delimitation-relevant than those covered here, due to the possibility that a state's continental shelf may, under certain conditions, extend beyond the 200-nm limit (which is strict for the EEZ). Such an extension is not automatic, however; it requires that a submission be made attesting that the continental margin of the state extends beyond 200 nm to the Commission on the Limits of the Continental Shelf (CLCS) and positive recommendation be procured from the said commission. Thus, a state that seeks to extend its continental shelf beyond 200 nm first needs to establish the right to do so and *delineate* these areas from the high seas, before proceeding to *delimiting* them with neighboring states that have likewise obtained the approval of the CLCS to extend their continental shelves. The ICJ, in its 1992 *St Pierre and Miquelon* judgment, refused French requests for a delimitation line to be drawn beyond 200-nm since the right to go beyond that limit first needs to be established through the procedure laid out in Article 76 of the UNCLOS (ICJ 1992, para. 82). The ICJ has also ruled that a state cannot claim an area beyond 200 nm if it means invading another state's claims within its 200-nm limit (see, ICJ's 2023 ruling in the *Nicaragua v. Colombia* case). The focus on delimitation-related disputes within the 200-nm limit is thus reasonable. If an increasing number of states are able to go beyond that limit, it will make sense to broaden the criterion for relevance from a reasonable year onwards—perhaps the second half of the first decade of the 2000s, when the initial deadline to make a submission to the CLCS to claim an extended continental shelf was set.

S2 Codebook

The dataset includes pairs of states with common maritime boundaries to delimit between 1946 and 2016. The description of the variables that are used to identify observations and those that are specific to this dataset are provided below. Other variables that are newly constructed or which are adapted from existing datasets are described before use in the main text and this supplement.

dyad A 4 to 6-digit identifier, composed of `statelno` and `statehno` as defined in the Correlates of War Project (COW) (Correlates of War Project 2017).[1]

ccode1 COW code for the first state, included first in the `dyad` code.

ccode2 COW code for the second state, making up the last three digits of the `dyad` code, preceded by zero if the state only has a two-digit code.

year Calendar year.

onsmdisp A maritime boundary dispute onset (0, 1).

amdisp There is an ongoing maritime boundary dispute (0, 1).

atdisp There is at least one related territorial dispute (0, 1).

status_num The state of maritime boundaries in a dyad according to whether (how much) they are delimited and disputed. There are five possible values:

- **1** Undelimited & Undisputed
- **2** Undelimited & Disputed
- **3** Partially delimited & Undisputed
- **4** Partially delimited & Disputed
- **5** Fully delimited & Undisputed

island_os There are offshore islands in the delimitation area (0, 1). This variable takes the value of 1 (a) where offshore island(s) of a state lies close to the mainland of another, or at least as far enough from its own principal coastline that taking it into account makes a considerable difference in its favor, and (b) the situation is asymmetric such that the latter state does not have similar offshore islands similarly situated. A typical example is the delimitation area in the Aegean Sea, where Greek islands are located close to the Turkish mainland. Note that island states (e.g., Malta, Saint Vincent and the Grenadines, Vanuatu) are considered to be mainlands.

They do not trigger a positive code for the `island_os` variable in their relations with other states. Island territories under the sovereignty of a state but with a certain degree of autonomy (e.g., Cook Islands, Cayman Islands, Greenland) and archipelagos (e.g., the Philippines) are assimilated to island-states and thereby also considered to be mainlands.

`adjacency` The coastal configuration of the delimitation area includes segments where coasts are adjacent to each other, as opposed to consisting of coasts that are uniquely opposite. For instance, Libya and Malta only have opposite coasts to delimit, so they would get 0 for this variable. Dyads that have only adjacent coasts to delimit (for example, Costa Rica and Panama, on both their Pacific and Atlantic coasts where their land boundaries meet) or coasts that are at least adjacent in some segments (for example, France and Italy in the area where their land borders meet, also having opposite coasts through various islands) are coded as 1. The classification of coastal configurations into adjacent, opposite, and mixed coasts was adopted from Tanaka (2004). The figure below, created in QGIS using the TS and EEZ boundaries mapped by Marine Regions (Flanders Marine Institute 2023), illustrates two examples of dyads that have adjacent coasts (left, between Angola and Namibia) and opposite coasts (right, between India and Sri Lanka).

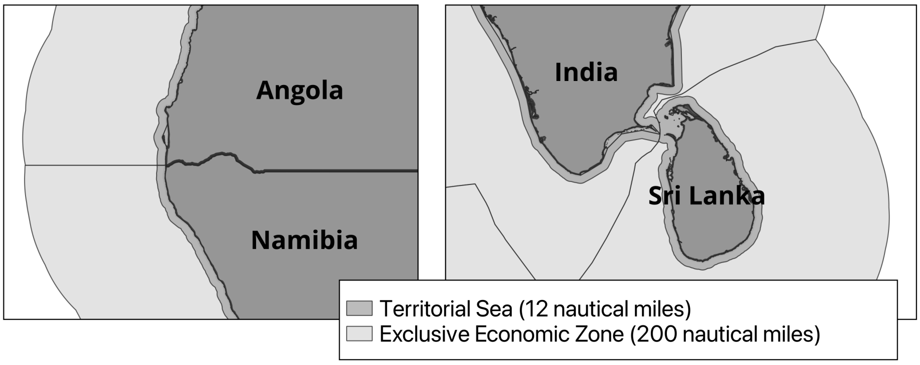

`cseez` There is a question of continental shelf and exclusive economic zone delimitation (0, 1). The variable takes the value of 0 if the maritime zones that have to be delimited are limited to the territorial sea and the contiguous zone (for instance, in the case of the maritime boundary between Croatia and Bosnia and Herzegovina). It takes the value of 1 if the question of delimitation beyond the territorial sea and the contiguous zone arises.

`petractive` Previous oil or gas discovery or production (0, 1). This variable takes the value of 1 from the moment in which there is a recorded oil discovery or production event, as coded by PETRODATA (Lujala et al. 2007), in the

area relevant to a dyadic boundary. If the area lies within 200 nm of both of the states, or it is no more than 250 nm from the point where the two states' land boundaries meet, the hydrocarbon activity is considered to be relevant to that dyad, and this variable takes the value of 1 from the year when there is oil discovery or production.

petrexpl Oil or gas exploration (0, 1). Constructed on the basis of `petractive`, this variable takes the value of 1 on the three years preceding an oil or gas discovery or production event.

region The region where the delimitation area is located. This is based on the original, 10-region classification used by the International Maritime Boundaries volumes (Charney 1993), which became 11 with the addition of the Caspian Sea (Colson 2005). I remove the Caspian Sea and instead add a "Multi-Region" category for when the boundary relations between two states span more than one region. The variable takes on the following values:

1 North America

2 Middle America and Caribbean

3 South America

4 Africa

5 Central Pacific and East Asia

6 Indian Ocean and South East Asia

7 Persian Gulf

8 Mediterranean and Black Sea

9 Northern and Western Europe

10 Baltic Sea

11 Caspian Sea

12 Multi-Region

S3 Descriptive tables and figures

S3.1 The state of dyadic maritime boundaries over time

Table S3.1 illustrates the variation in the dataset in terms of where dyads stand with regard to the dispute and delimitation status of their common maritime boundaries in 10-year intervals. By the end of the time period under observation, there are almost as many dyads that have their boundaries fully delimited (130) as those that have not yet any delimitation or dispute (139).

Boundary Status	1946	1956	1966	1976	1986	1996	2006	2016
Undelim. & Undisp.	65 (0.82)	135 (0.89)	207 (0.87)	199 (0.69)	191 (0.60)	178 (0.53)	160 (0.47)	139 (0.41)
Undelim. & Disp.	6 (0.08)	8 (0.05)	10 (0.04)	32 (0.11)	38 (0.12)	40 (0.12)	33 (0.10)	36 (0.11)
Part. Delim. & Undisp.	8 (0.10)	9 (0.06)	18 (0.08)	39 (0.14)	23 (0.07)	26 (0.08)	24 (0.07)	19 (0.06)
Part. Delim. & Disp.	0 (0.00)	0 (0.00)	3 (0.01)	4 (0.01)	14 (0.04)	15 (0.04)	15 (0.04)	13 (0.04)
Full. Delim. & Undisp.	0 (0.00)	0 (0.00)	1 (0.00)	14 (0.05)	52 (0.16)	78 (0.23)	105 (0.31)	130 (0.39)
Total dyads in dispute	6 (0.08)	8 (0.05)	13 (0.05)	36 (0.12)	52 (0.16)	55 (0.16)	48 (0.14)	49 (0.15)
Total dyads	79 (1.00)	152 (1.00)	239 (1.00)	288 (1.00)	318 (1.00)	337 (1.00)	337 (1.00)	337 (1.00)

Table S3.1: Distribution of dyads at ten-year intervals according to their boundary status. The share of a given group of dyads in that year is reported as a proportion in parentheses. Note: (Un)delim. = (Un)delimited; Part./Full. = Partially/Fully; (Un)disp. = (Un)disputed.

S3.2 Summary statistics of the numerical variables used in models

What follows in Table S3.2 are summary statistics of the main numerical variables. I present these for the subset of dyads at risk of observing a dispute, which is the part of the data used in the statistical tests.

Statistic	N	Mean	St. Dev.	Min	Max
Maritime boundary dispute onset	15,776	0.007	0.085	0	1
Offshore island	15,776	0.306	0.461	0	1
Adjacency	15,776	0.434	0.496	0	1
Beyond territorial sea	15,776	0.898	0.303	0	1
Number of dyadic boundaries to delimit	15,776	12.656	8.323	2	59
Hydrocarbon exploration	11,530	0.051	0.219	0	1
Hydrocarbon activity	13,962	0.325	0.469	0	1
Existing related territorial dispute	15,776	0.233	0.423	0	1
Existing maritime boundary dispute	15,776	0.155	0.362	0	1
Capability ratio	14,205	0.831	0.150	0.500	1.000
Total catch (logged)	14,720	12.847	2.080	5.011	16.931

Table S3.2: Descriptive statistics of the main numerical variables.

S4 Marginal effects including controls

Figures S4.1 and S4.2 present the full set of marginal effects based on models presented in Tables 2 and 3 in the main text.

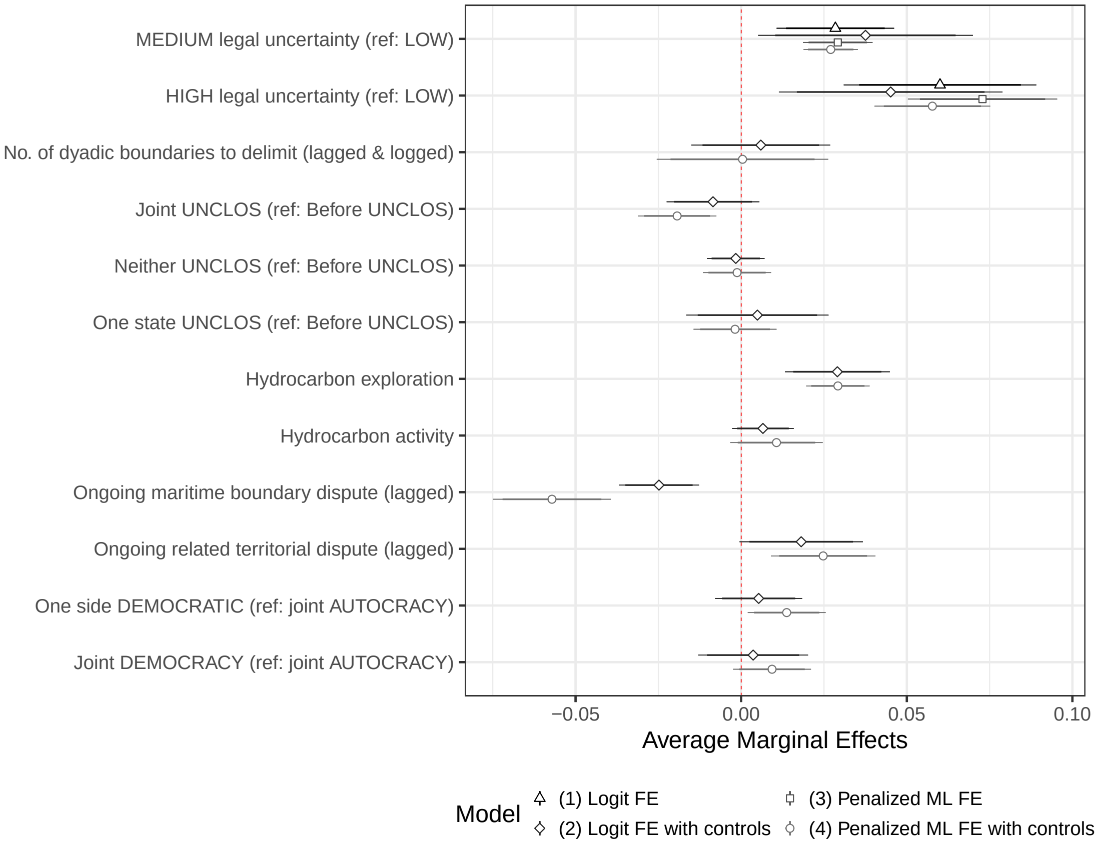

Figure S4.1: Average marginal effects on the probability of dispute onset.

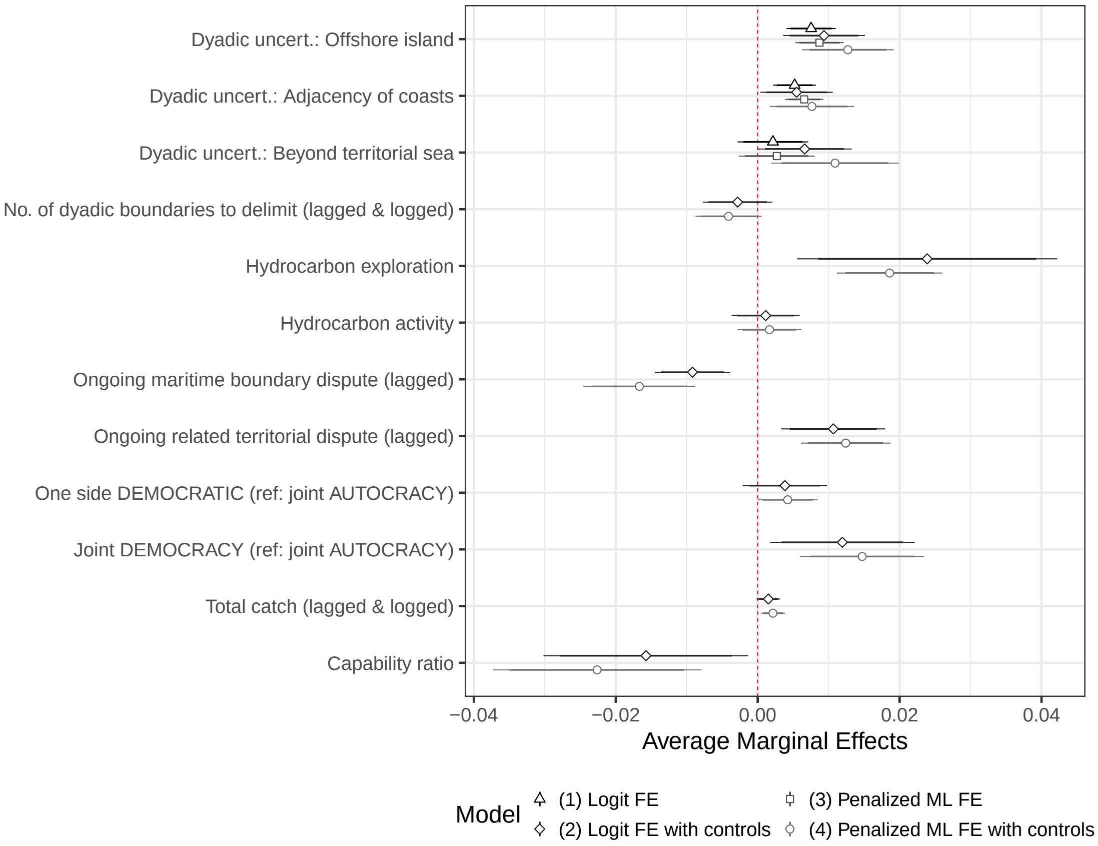

Figure S4.2: Average marginal effects on the probability of dispute onset.

S5 Robustness checks

S5.1 Alternative period variables

Table S5.1 present regression output with the inclusion of alternative period variables (capturing UNCLOS-I, UNCLOS-II, UNCLOS-III negotiations; the signature and entry into force of UNCLOS, and post-Cold War).

	Model 1	Model 2	Model 3	Model 4	Model 5	Model 6
MEDIUM legal uncertainty (ref: LOW)	2.41	1.73***	0.43	0.38	2.20	1.53**
	(1.23)	(0.43)	(0.79)	(0.40)	(1.23)	(0.48)
HIGH legal uncertainty (ref: LOW)	2.83*	2.19***	1.20	1.17*	3.07*	2.35***
	(1.26)	(0.58)	(0.91)	(0.59)	(1.25)	(0.63)
No. of dyadic boundaries to delimit (lagged & logged)	0.66	0.06	0.51	0.01	0.59	−0.01
	(1.50)	(0.68)	(1.58)	(0.73)	(1.53)	(0.68)
Joint UNCLOS (ref: Before UNCLOS)	−1.06	−0.93			−1.23	−1.10
	(1.92)	(0.86)			(1.96)	(0.83)
Neither UNCLOS (ref: Before UNCLOS)	−0.05	0.04			−0.18	−0.05
	(0.66)	(0.68)			(0.68)	(0.65)
One state UNCLOS (ref: Before UNCLOS)	0.71	0.13			0.49	−0.02
	(1.20)	(0.69)			(1.22)	(0.68)
Hydrocarbon exploration	1.74**	1.34***	1.76**	1.34***	1.75**	1.35***
	(0.59)	(0.21)	(0.64)	(0.22)	(0.60)	(0.22)
Hydrocarbon activity	0.53	0.41	0.44	0.40	0.63	0.49
	(0.62)	(0.26)	(0.61)	(0.27)	(0.62)	(0.27)
Ongoing maritime boundary dispute (lagged)	−3.01**	−2.63***	−2.83**	−2.40***	−3.01**	−2.59***
	(0.92)	(0.65)	(0.87)	(0.67)	(0.94)	(0.67)
Ongoing related territorial dispute (lagged)	1.27	0.99	1.70	1.26*	1.49	1.17*
	(1.01)	(0.56)	(0.99)	(0.57)	(1.02)	(0.54)
One side DEMOCRATIC (ref: joint AUTOCRACY)	0.34	0.45	0.16	0.32	0.39	0.52
	(1.08)	(0.32)	(1.11)	(0.35)	(1.13)	(0.32)
Joint DEMOCRACY (ref: joint AUTOCRACY)	0.45	0.60	0.29	0.48	0.52	0.67
	(0.85)	(0.24)	(0.88)	(0.25)	(0.87)	(0.23)
UNCLOS negotiations	0.68	0.54				
	(0.48)	(0.42)				
UNCLOS signed			−0.23	−0.16		
			(0.71)	(0.65)		
UNCLOS in force			−4.16*	−3.31***		
			(2.08)	(0.73)		
Post-Cold War					−0.35	−0.39
					(1.08)	(0.68)
Dyad-fixed effects	Yes	Yes	Yes	Yes	Yes	Yes
Year-fixed effects	No	No	No	No	No	No
Cubic polynomial for time	Yes	Yes	Yes	Yes	Yes	Yes
Num. obs.	2229	2229	2229	2229	2229	2229
Num. groups: dyad	63	63	63	63	63	63

***$p < 0.001$; **$p < 0.01$; *$p < 0.05$ Robust standard errors clustered by dyad in parentheses.

Table S5.1: Regression output for robustness checks with alternative period variables.

Figures S5.1 and S5.2 present average marginal effects from the same six models, presented this way in two figures for the sake of legibility.

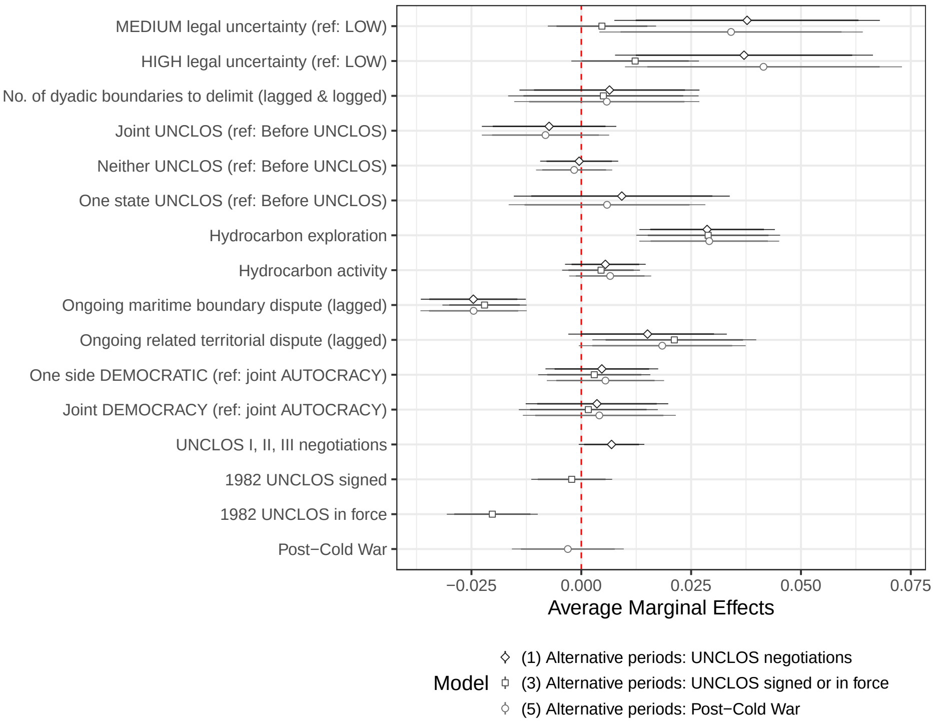

Figure S5.1: Average marginal effects with alternative period variables (FE Logit models).

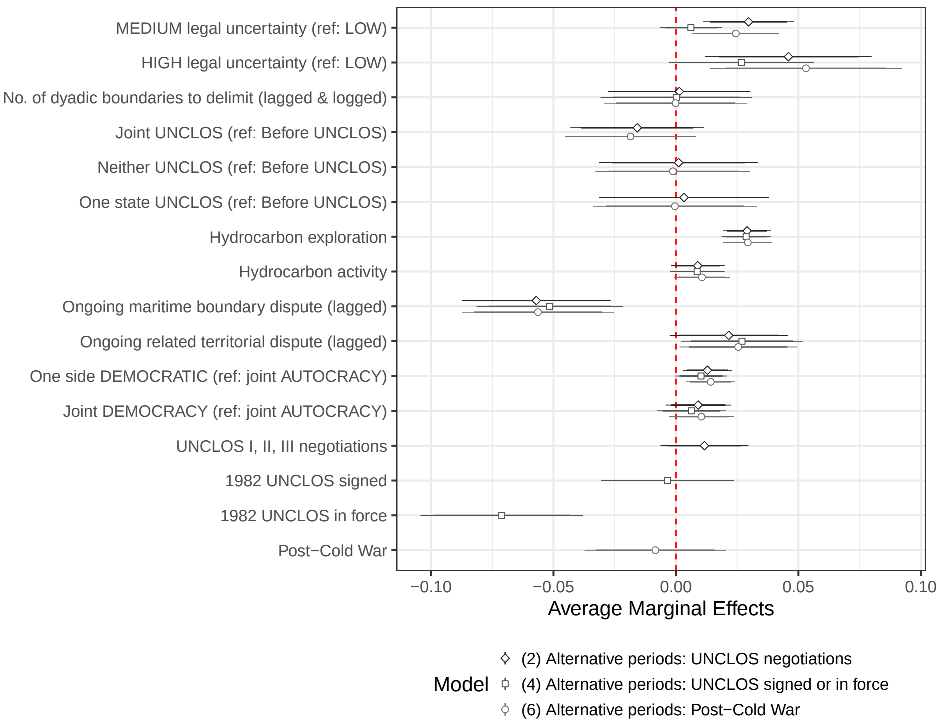

Figure S5.2: Average marginal effects with alternative period variables (Penalized ML models).

S5.2 Legal origin and recent independence

The following considers whether the fact that states differ in their domestic legal systems (as defined in Powell and Mitchell (2007)) or that some states have joined the system more recently than others (typically, in periods of medium and high legal uncertainty) may be driving some of the variation in dispute onset.

	FE Logit	Penalized ML
Offshore island	0.99***	0.95***
	(0.29)	(0.24)
Adjacency of coasts	0.63*	0.61*
	(0.27)	(0.21)
Beyond territorial sea	1.12	0.78
	(1.03)	(0.35)
No. of dyadic boundaries to delimit (lagged & logged)	−0.35	−0.35
	(0.31)	(0.19)
Hydrocarbon exploration	1.47***	1.42***
	(0.37)	(0.29)
Hydrocarbon activity	0.11	0.11
	(0.27)	(0.17)
Total catch (lagged & logged)	0.15	0.15*
	(0.10)	(0.06)
Capability ratio	−1.55+	−1.55*
	(0.82)	(0.58)
Ongoing maritime boundary dispute (lagged)	−1.44**	−1.37***
	(0.50)	(0.29)
Ongoing related territorial dispute (lagged)	1.05***	1.02***
	(0.28)	(0.24)
One side DEMOCRATIC (ref: joint AUTOCRACY)	0.80+	0.79*
	(0.42)	(0.29)
Joint DEMOCRACY (ref: joint AUTOCRACY)	0.28	0.28
	(0.31)	(0.20)
Same domestic legal origin	−0.18	−0.17
	(0.31)	(0.20)
One state independence after 1946 (ref: Both independent before 1946)	−0.66	−0.62*
	(0.43)	(0.31)
Both states independence after 1946 (ref: Both independent before 1946)	−0.25	−0.23
	(0.47)	(0.32)
Dyad-fixed effects	No	No
Year-fixed effects	Yes	Yes
Cubic polynomial for time	Yes	Yes
Num. obs.	4516	4516
Num. groups: year	28	28

***$p < 0.001$; **$p < 0.01$; *$p < 0.05$; $^{+}p < 0.1$
Robust standard errors clustered by year in parentheses.

Table S5.2: Statistical models assessing the robustness of main results about the relationship between dyad-specific uncertainty and dispute onset.

Figure S5.3 presents associated marginal effects.

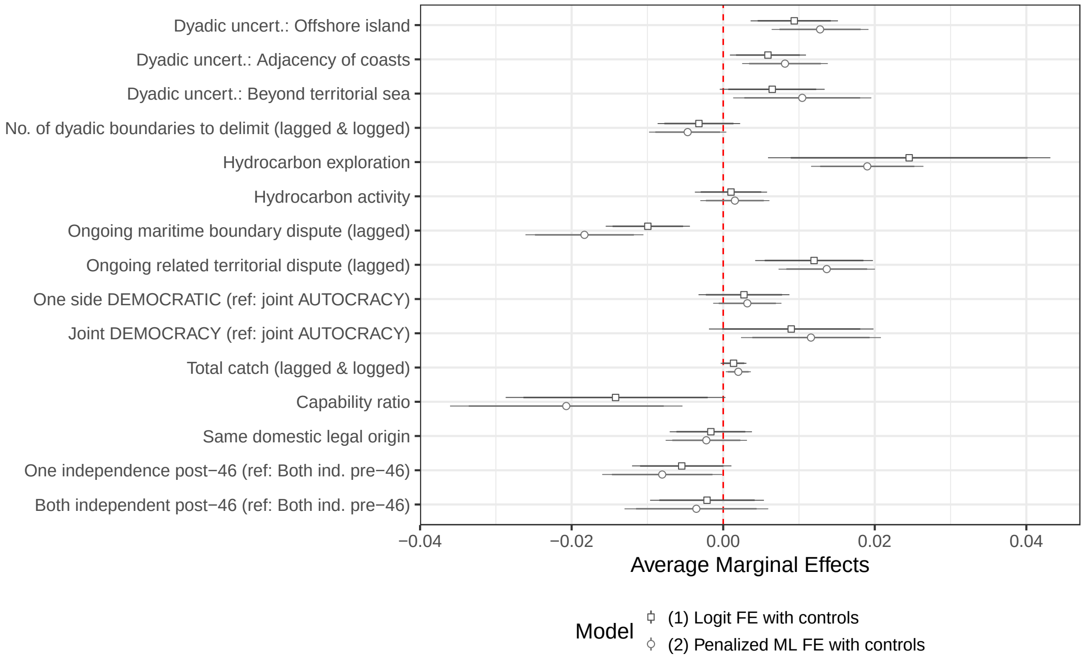

Figure S5.3: Average marginal effects on the probability of dispute onset.

S5.3 The relation between dyadic and baseline uncertainty

If baseline uncertainty and dyad-specific uncertainty work together, we should expect dyad-specific uncertainty factors to be more strongly associated with dispute onset if baseline uncertainty is also high. To probe this, I employ a dynamic difference-in-differences strategy (Sun and Abraham 2021), considering dyad-specific uncertainty factors to be treatments that are present once baseline legal uncertainty is high (from 1969 onwards).

For ease of interpretation, I divide up the years into eight periods of eight years each, and consider treatment to begin from the third period onwards (1969-1976). I fit four OLS models, with the following regression equation:

$$\text{Onset} = \alpha_i + \gamma_t + \sum_{\substack{k=1\\k\neq 2}}^{8} \beta_{P_k} \text{Treated} + \varepsilon_{it} \tag{1}$$

where α_i are fixed effects for whether a dyad is in a group that receives the treatment or not, γ_t are fixed effects for period, Treated takes the value of 1 if the dyad has a dyadic uncertainty factor, and β_{P_k} are coefficients for periods before and after treatment. The coefficient for the period just before the treatment (β_{P_2}) is omitted and used as the baseline.

The first model considers there to be dyad-specific uncertainty if a dyad has at least one of the dyad-specific factors. The second, third, and fourth models focus on each dyad-specific factor separately: adjacency, delimitation beyond territorial sea, and offshore islands. The average treatment effect on the treated (ATT) is calculated based on these models and presented in Figure S5.4.

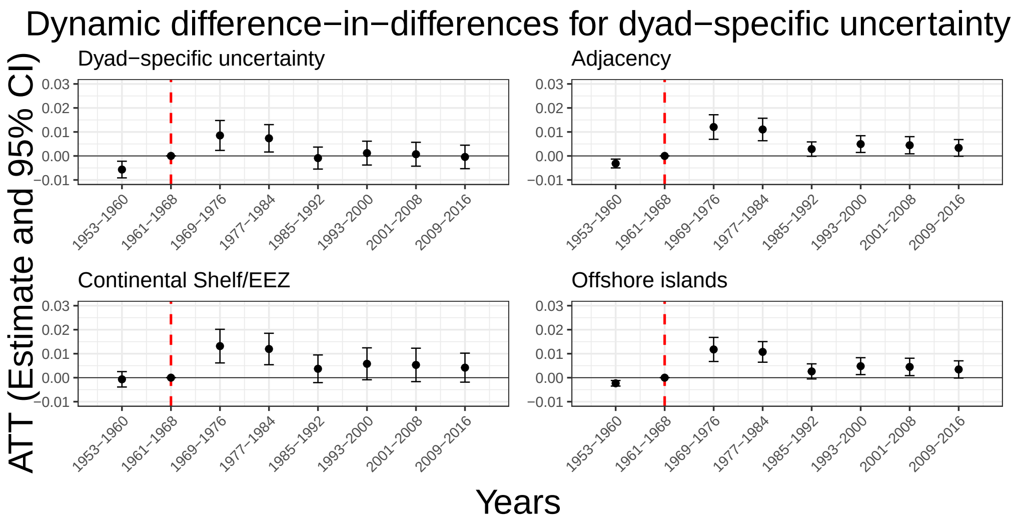

Figure S5.4: Average treatment effect on the treated, calculated based on difference-in-differences models for each pre- and post-treatment period.

The results suggest that dyadic factors of uncertainty contribute more to dispute onset when baseline uncertainty is also high (in the two first periods after treatment).

S5.4 Regional patterns of dispute onset and legal uncertainty

Legal uncertainty could operate differently in different regions—it can be stronger in some, or quicker to have an effect. This section considers this possibility.

Figure S5.5 depicts the number of disputes beginning in five groups of regions, constructed on the basis of the **region** variable (see the Codebook, section S2 of this OS).

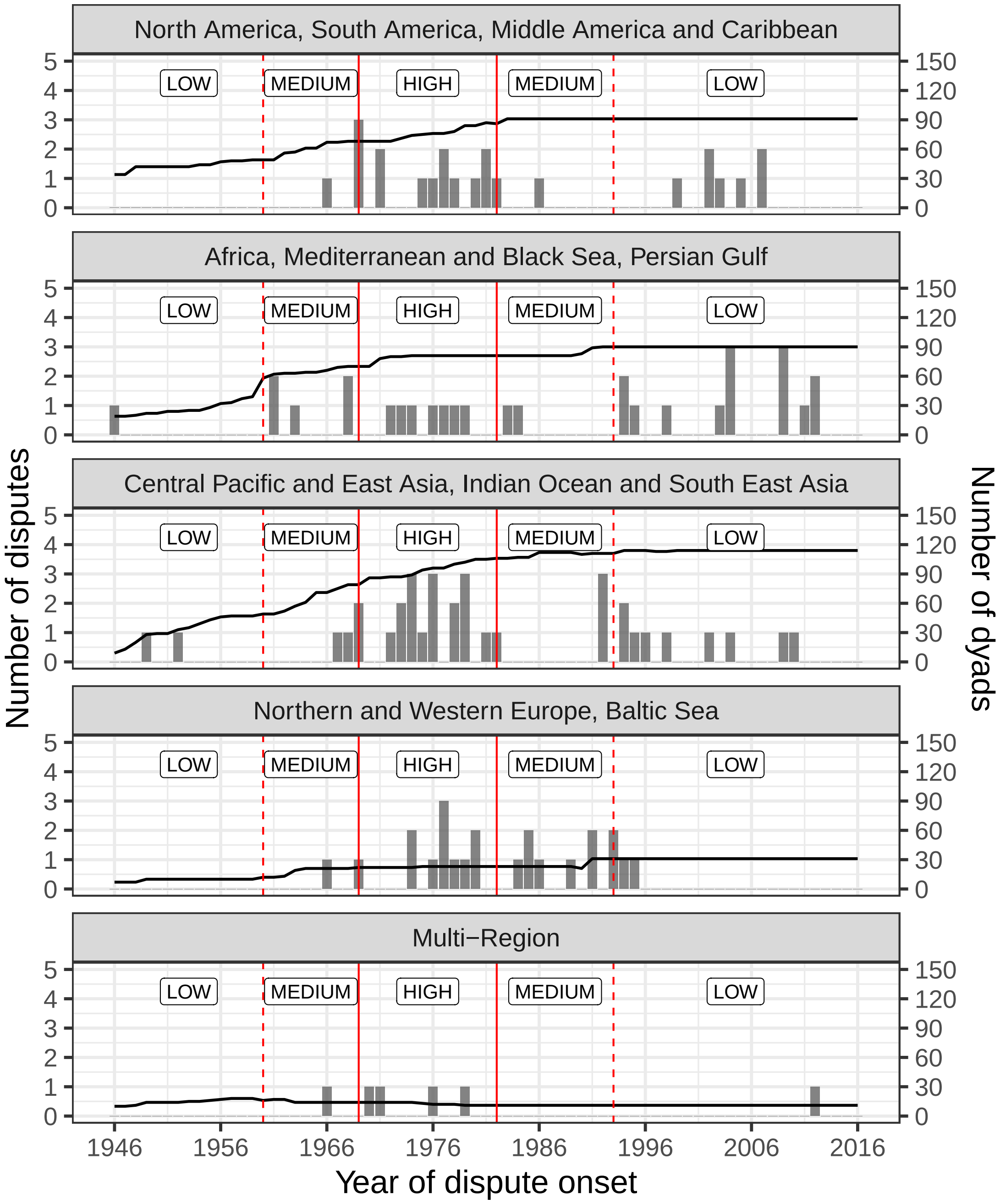

Figure S5.5: Number of disputes and dyads in five region groups, with levels of baseline legal uncertainty overlaid. The Multi-Region category consists of dyads which have relations in more than one of the 10 regions defined in the Codebook.

While there are clear differences across regions, the period with high baseline legal uncertainty is the one in which dispute activity is greater than average. The second group (Africa, Mediterranean and Black Sea, and the Persian Gulf) is an exception, with several disputes beginning in later years, when legal uncertainty is low. The fourth group, consisting of Northern and Western Europe and Baltic Sea fits best with what the theory expects.

Table S5.3 and Figure S5.6 present the regression output and marginal effects calculated on the basis of a series of regressions that examine the relation between legal uncertainty (in both its systemic and dyadic component) and dispute onset in each region group.

	Region Group 1		Region Group 2		Region Group 3		Region Group 4	
	Model 1	Model 2	Model 3	Model 4	Model 5	Model 6	Model 7	Model 8
MEDIUM legal uncertainty (ref: LOW)	1.90***		0.57		0.79*		4.00***	
	(0.43)		(0.23)		(0.29)		(1.13)	
HIGH legal uncertainty (ref: LOW)	2.88***		0.79+		1.98***		4.66***	
	(0.53)		(0.27)		(0.37)		(1.21)	
Offshore islands		1.32***		0.79**		1.08***		0.20
		(0.21)		(0.18)		(0.22)		(0.18)
Adjacency		0.75*		0.61+		0.58*		0.25
		(0.21)		(0.19)		(0.21)		(0.29)
Beyond territorial sea		−0.89+		−0.31		1.33*		−0.10
		(0.26)		(0.38)		(0.28)		(0.12)
Dyad-fixed effects	Yes	No	Yes	No	Yes	No	Yes	No
Year-fixed effects	No	Yes	No	Yes	No	Yes	No	Yes
Cubic polynomial for time	Yes	Yes	Yes	Yes	Yes	Yes	Yes	Yes
Num. obs.	4414	4414	4123	4123	5480	5480	1040	1040

$^{***}p < 0.001$; $^{**}p < 0.01$; $^{*}p < 0.05$; $^{+}p < 0.1$

Table S5.3: Statistical models for baseline and dyad-specific uncertainty in different regional groups.

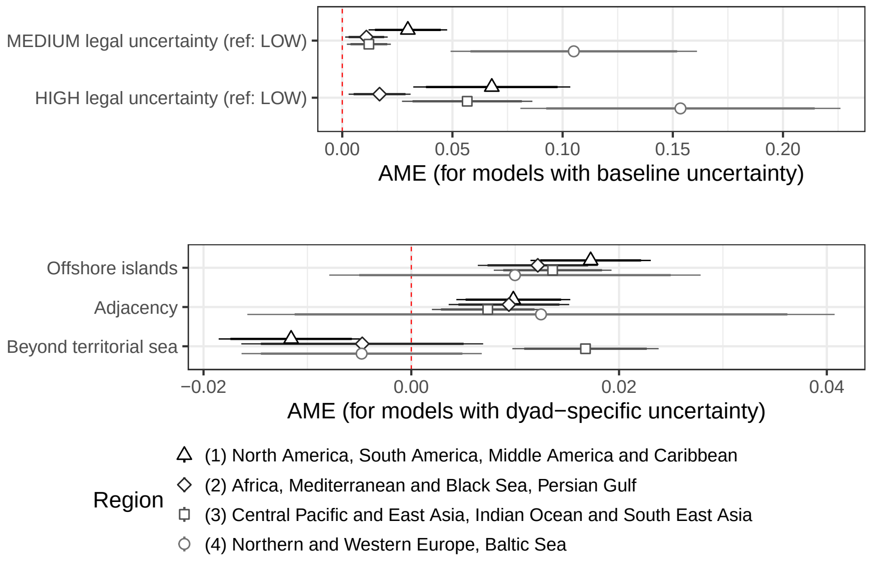

Figure S5.6: Average marginal effects calculated on the basis of regressions with baseline and dyad-specific uncertainty, run separately for different region groups.

Looking first at baseline uncertainty, we observe that the strength of the relationship between baseline uncertainty and onset is greater in the region group 4 (Northern and Western Europe, Baltic Sea) and weaker in the region group 2 (Africa, Mediterranean and Black Sea, Persian Gulf)), consistent with Figure S5.5. There is also a good deal of variation in the expected change in probability for facing MEDIUM or HIGH uncertainty as opposed to LOW levels of uncertainty. HIGH uncertainty has the greater marginal effects overall, reaching as far as 0.15 in one region group.

When it comes to the dyad-specific uncertainty, offshore islands are strong predictors of disputes in all, followed by adjacency. The need for delimiting beyond the territorial seems to be a possible driver of dispute in only the region group 3, consisting of Central Pacific and East Asia, Indian Ocean and South East Asia.

While it is beyond the scope of this paper to uncover the sources of this heterogeneity across regions, we can suggest that the maritime boundary-making activity is clustered not only in time but also geographically, and the fact that some regions experience dispute activity later (when the waters are calmer in others) can be due to a lack of interest in drawing maritime boundaries in earlier periods.

S5.5 Legal uncertainty vs. legal void

The period until 1960 was coded as having a low level of legal uncertainty, based on the definition of legal uncertainty offered in this paper. It may be argued, however, there was instead a period of legal void in the lead-up to 1958 (UNCLOS-I), because the extent of state jurisdiction in the sea was not defined and state practice was limited. This period of legal void may still drive disputes by giving way to new state practice that many states could object to. It may also be that legal gaps prevent disputes by keeping excessive claims at bay as states default to customary rules of limited state jurisdiction. To assess these possibilities, I run two models with only the baseline legal uncertainty variable as well as the indicator for legal void. The main findings about baseline legal uncertainty hold. Legal void, for its part, also appears associated with dispute onset, controlling for legal uncertainty. Table S5.4 presents the output and Figure S5.7 the average marginal effects.

	Model 1	Model 2
Legal void	2.18*	1.95***
	(0.89)	(0.65)
MEDIUM legal uncertainty (ref: LOW)	2.21***	1.94***
	(0.57)	(0.43)
HIGH legal uncertainty (ref: LOW)	3.32***	2.92***
	(0.68)	(0.38)
Dyad-fixed effects	Yes	Yes
Year-fixed effects	No	No
Cubic polynomial for time	Yes	Yes
Num. obs.	4672	4672
Num. groups: dyad	103	103

$^{***}p < 0.001$; $^{**}p < 0.01$; $^{*}p < 0.05$; $^{+}p < 0.1$

Robust standard errors clustered by dyad in parentheses.

Table S5.4: Separately testing for legal void.

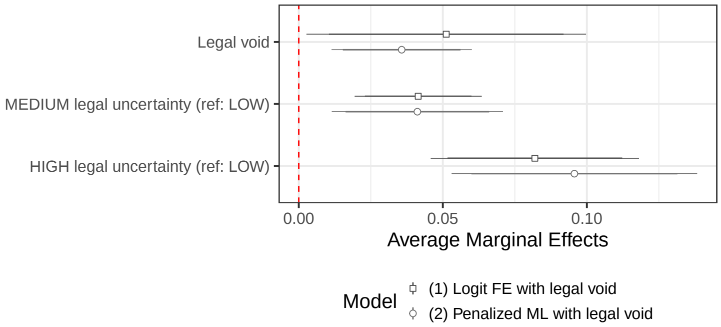

Figure S5.7: Average marginal effects of legal void and baseline legal uncertainty on the probability of dispute onset.

S5.6 Number of disputes and legal uncertainty

I run two count models—a Poisson regression and a negative binomial regression—with data aggregated so that we have 71 observations representing the years between 1946 and 2016. I use the baseline legal uncertainty as the main predictor, and use the controls described in Table S5.5.

Statistic	N	Mean	St. Dev.	Min	Max
Year	71	1,981.000	20.640	1,946	2,016
Number of onsets	71	1.634	1.838	0	7
Number of onsets in the preceding year	70	1.657	1.841	0	7
Cumulative number of onsets	71	54.930	42.675	1	116
Change in the number of dyads	70	3.686	4.871	−2	21
Proportion of dyads with joint UNCLOS	71	0.208	0.300	0.000	0.745
Cumulative number of fully delimited boundaries	70	44.186	44.373	0	129

Table S5.5: Summary statistics for the variables used in the count models.

The coefficients estimated based on the two regressions and average marginal effects are reported in Table S5.6.

	Poisson		Negative Binomial	
	Coefficients	Marginal effects	Coefficients	Marginal effects
MEDIUM legal uncertainty (ref: LOW)	0.89*	1.18	0.91*	1.21
	(0.40)	(0.63)	(0.45)	(0.72)
HIGH legal uncertainty (ref: LOW)	1.60***	3.25**	1.61**	3.28*
	(0.43)	(1.25)	(0.50)	(1.53)
Change in the number of dyads	−0.03	−0.04	−0.02	−0.04
	(0.03)	(0.05)	(0.04)	(0.06)
Proportion of dyads with joint UNCLOS	0.64	1.05	0.47	0.77
	(1.16)	(1.91)	(1.36)	(2.26)
Number of onsets in the preceding year	0.08	0.13	0.09	0.15
	(0.06)	(0.10)	(0.08)	(0.13)
Cumulative number of fully delimited boundaries	0.00	0.00	0.00	0.01
	(0.01)	(0.01)	(0.01)	(0.01)
Constant	−0.52		−0.62	
	(0.40)		(0.45)	
AIC	235.89		234.39	
BIC	251.63		252.38	
Log Likelihood	-110.95		-109.20	
Num. obs.	70		70	

$^{***}p < 0.001$; $^{**}p < 0.01$; $^{*}p < 0.05$

Table S5.6: Regression output of count models with the number of disputes in a given year as the dependent variable.

S6 Figures and tests probing the mechanism

Figure S6.1 depicts the proportion of matching dyadic limits and delimitation methods over time, with the period of high baseline uncertainty depicted using the dashed vertical lines. The matches in question are between the unilateral claims made by each state in the dyad as to (a) the outer limits of their territorial seas and fishing zones/EEZ, (b) the definition of their continental shelf, (c) the preferred method for delimiting the continental shelf. We observe a clear break in the matches in the fishing zone/EEZ outer limits, the definition of the CS, and the preferred CS delimitation method.

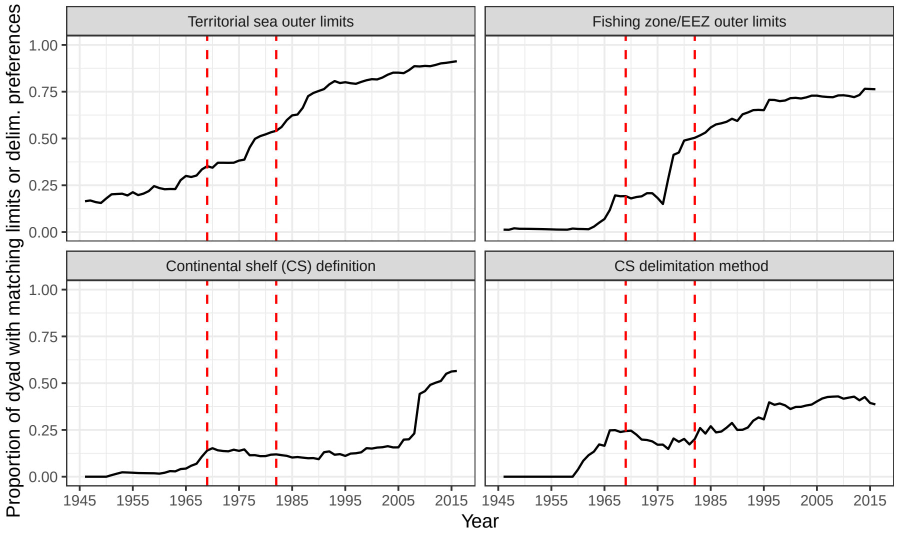

Figure S6.1: Matches in outer limits and delimitation method preferences.

Figure S6.2 presents average marginal effects based on Table 4, reported in the main text. When legal uncertainty is high, the probability of having matching policies is substantively lower for all types of unilateral claims considered.

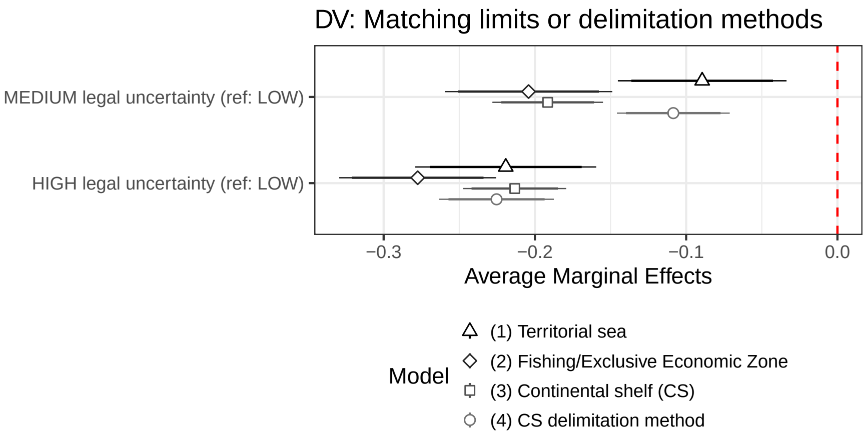

Figure S6.2: Average marginal effects of uncertainty on matching.

Table S6.1 present regression output for two logit models with dyad-fixed effects with dispute onset as the outcome variable. The models control for baseline legal uncertainty, and focus on the relationship between different combinations of matches in limits and methods on the one hand and dispute onset on the other hand.

	DV: Dispute onset	
	FE Logit	Penalized ML
MEDIUM legal uncertainty (ref: LOW)	2.32^{**}	1.99^{***}
	(0.75)	(0.28)
HIGH legal uncertainty (ref: LOW)	3.32^{***}	2.88^{***}
	(0.84)	(0.34)
Neither limits nor methods match (ref: Both match)	2.06^{*}	1.83^{***}
	(0.81)	(0.30)
Only limits match (ref: Both match)	1.64^{+}	1.53^{***}
	(0.85)	(0.34)
Only methods match (ref: Both match)	0.19	0.26
	(0.78)	(0.23)
Dyad-fixed effects	Yes	Yes
Year-fixed effects	No	No
Cubic polynomial for time	Yes	Yes
Num. obs.	4219	4219
Num. groups: dyad	95	95

$^{***}p < 0.001$; $^{**}p < 0.01$; $^{*}p < 0.05$; $^{+}p < 0.1$

Robust standard errors clustered by dyad in parentheses.

Table S6.1: The relation between matching limits and delimitation method preferences and dispute onset, controlling for baseline legal uncertainty. Fixed effects and cubic polynomial terms are omitted.

Notes

[1]Some state codes needed to be added to the original set of states listed by the COW Project: 923 for Cook Islands and 926 for Niue Both are self-governing states in free association with New Zealand. Niue already had 926 assigned to it in the COW Colonial Contiguity Dataset (Correlates of War Project 2021).

References

Charney, J. I. (1993). Introduction. In J. I. Charney and L. M. Alexander (Eds.), *International Maritime Boundaries*, Volume I, pp. xxiii–xlvi. Dordrecht: Martinus Nijhoff Publishers.

Colson, D. A. (2005). Introduction. In D. A. Colson and R. W. Smith (Eds.), *International Maritime Boundaries*, Volume V, pp. xxvii–xxxi. Leiden and Boston: Martinus Nijhoff Publishers.

Correlates of War Project (2017). State system membership list, v2016.

Correlates of War Project (2021). Colonial contiguity data, 1816-2016. version 3.1.

Flanders Marine Institute (2023). Maritime boundaries geodatabase: Maritime boundaries and exclusive economic zones (200nm), version 12. Available online at https://www.marineregions.org/.

Lujala, P., J. K. Rød, and N. Thieme (2007). Fighting over Oil: Introducing a New Dataset. *Conflict Management and Peace Science 24*(3), 239–256.

Powell, E. J. and S. M. Mitchell (2007). The International Court of Justice and the World's Three Legal Systems. *The Journal of Politics 69*(2), 397–415.

Sun, L. and S. Abraham (2021). Estimating dynamic treatment effects in event studies with heterogeneous treatment effects. *Journal of Econometrics 225*(2), 175–199.

Tanaka, Y. (2004). Reflections on Maritime Delimitation in the Cameroon / Nigeria Case. *The International and Comparative Law Quarterly 53*(2), 369–406.
